# Selective Photonic Gasification of Strained Oxygen Clusters on Graphene for Tuning Pore Size in the Å Regime

**DOI:** 10.1021/jacsau.3c00395

**Published:** 2023-09-29

**Authors:** Luc Bondaz, Anshaj Ronghe, Shaoxian Li, Kristia̅ns Čerņevičs, Jian Hao, Oleg V. Yazyev, K. Ganapathy Ayappa, Kumar Varoon Agrawal

**Affiliations:** † Laboratory of Advanced Separations, Institute of Chemical Sciences & Engineering, École Polytechnique Fédérale de Lausanne (EPFL), CH-1950 Sion, Switzerland; ‡ Department of Chemical Engineering, Indian Institute of Science, Bangalore 560012, India; § Institute of Physics, EPFL, Lausanne CH-1015, Switzerland

**Keywords:** porous graphene, oxygen cluster, gasification, pore formation, membranes, gas separation, molecular dynamics

## Abstract

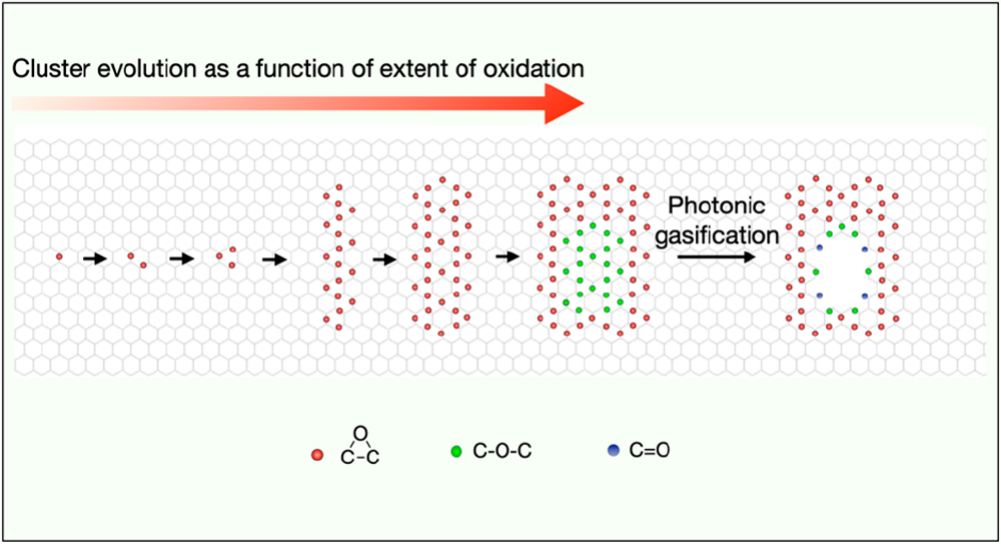

Controlling the size of single-digit pores, such as those in graphene, with an Å resolution has been challenging due to the limited understanding of pore evolution at the atomic scale. The controlled oxidation of graphene has led to Å-scale pores; however, obtaining a fine control over pore evolution from the pore precursor (i.e., the oxygen cluster) is very attractive. Herein, we introduce a novel “control knob” for gasifying clusters to form pores. We show that the cluster evolves into a core/shell structure composed of an epoxy group surrounding an ether core in a bid to reduce the lattice strain at the cluster core. We then selectively gasified the strained core by exposing it to 3.2 eV of light at room temperature. This allowed for pore formation with improved control compared to thermal gasification. This is because, for the latter, cluster–cluster coalescence via thermally promoted epoxy diffusion cannot be ruled out. Using the oxidation temperature as a control knob, we were able to systematically increase the pore density while maintaining a narrow size distribution. This allowed us to increase H_2_ permeance as well as H_2_ selectivity. We further show that these pores could differentiate CH_4_ from N_2_, which is considered to be a challenging separation. Dedicated molecular dynamics simulations and potential of mean force calculations revealed that the free energy barrier for CH_4_ translocation through the pores was lower than that for N_2_. Overall, this study will inspire research on the controlled manipulation of clusters for improved precision in incorporating Å-scale pores in graphene.

## Introduction

The zero-dimensional nature of Å-scale pores in graphene makes them attractive for exploring and manipulating molecular transport^1−4^ at the atomic scale for applications in molecular and ionic separation,^2,5−12^ sensing,^13,14^ molecular valves,^15^ and phase transitions under confinement.^8,16,17^ In particular, the possibility to finely tune the pore size and pore density in graphene is highly sought after for advanced applications in gas- and liquid-phase separation.^5,10,18,19^ Computational studies on molecular transport across porous graphene have reported highly attractive separation performances.^20−24^ Motivated by these results, efforts have been made to achieve Å-scale pores with a narrow size distribution by several physical and chemical routes. These include direct carbon knockout by focused ion beam techniques,^25,26^ electron beam methods,^27^ and plasma procedures,^28−31^ as well as carbon gasification through chemical etching using KMnO_4_,^32^ O_3_,^6,33^ O_2_,^6^ and a combination of these.^2,28,32^ Oxidation chemistry is extremely attractive for pore formation because of its high uniformity and ease of implementation, which has resulted in the commercialization of oxidized graphene in the form of graphene oxide (GO) and reduced GO (rGO).^34^ However, in order to achieve a fine control over the size distribution in the Å regime of graphene, one must improve the understanding of the underlying mechanism for pore formation.^35^

**Scheme 1:**
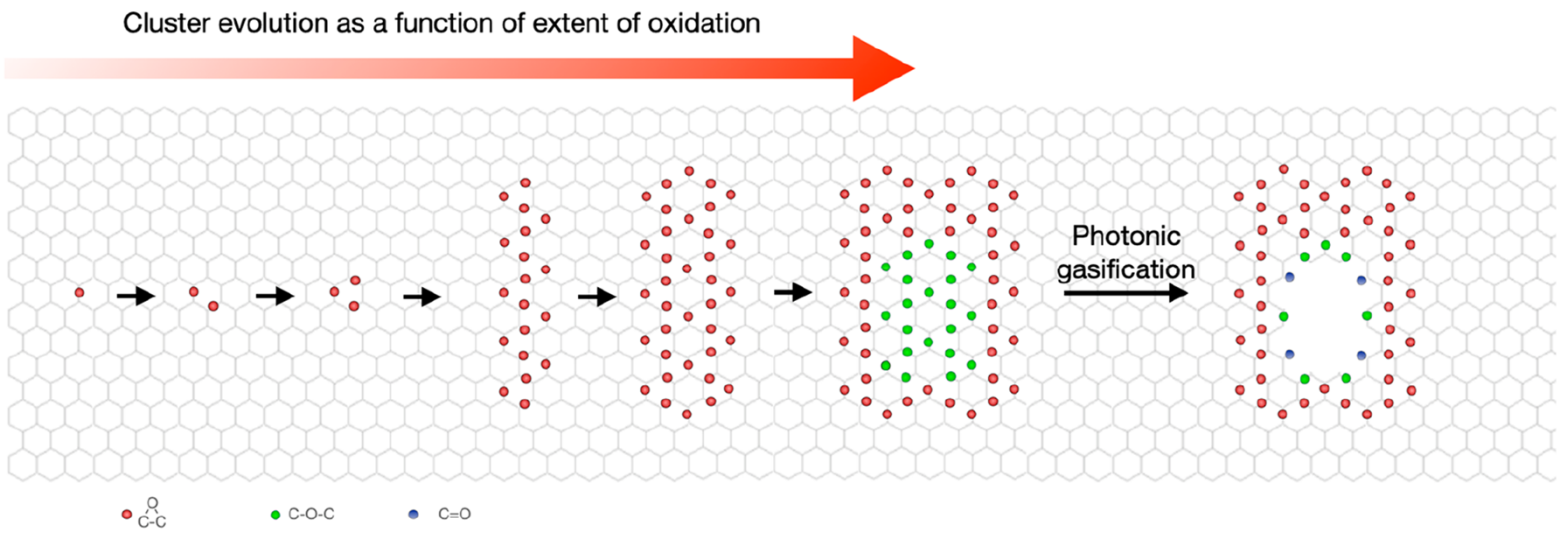
Oxygen Cluster Evolution and Formation of Pores by Gasification Upon ozone exposure, the oxygen clusters grow, forming a core/shell structure that is created by an epoxy shell circling an ether core. Ultimately, exposure to light gasifies the strained cluster core.

The following fundamental understandings are crucial: identification of precursors which evolve into vacancy defects or pores; understanding how precursor properties (e.g., chemical composition, structure, and size) are related to that of the pores; and understanding the energetics of the underlying processes (i.e., precursor nucleation and growth and the conversion of precursor to pore).^35,36^ Progress has been achieved in some of these aspects. For example, it is now well understood that epoxy groups are generated on the graphitic lattice upon oxidation. They form energy-minimizing clusters, which are helped by their low barrier for diffusion (0.7 eV) on the graphitic lattice, that subsequently bind together in order to reduce the net energy of the system.^6^ Clusters as small as epoxy dimers serve as stable nuclei for further growth into larger clusters.^37^ These clusters grow as cyclic epoxy trimers, starting out as linear chains that then coalesce to form larger clusters where the trimers are organized in a honeycomb superstructure (Scheme 1). In situ aberration-corrected transmission electron microscopy (AC-HRTEM) experiments have revealed that the clusters gasify to form pores with an energy barrier of 1.3 eV, and the pore size is limited by the size of the cluster.^37^

Spectroscopic evidence has shown that ether groups manifest within the large epoxy clusters; however, their exact location has not been pinpointed.^37^ Additionally, the role of the ether group in the gasification process and the resulting pore size has remained unclear. Herein, we demonstrate that the ether group plays a critical role in regulating the size of the pore within the oxygen cluster. We establish the fact that ether groups are formed in the core of the epoxy cluster, driven by the higher strain at the core, which facilitates the cleavage of the C–C bond. We show that the formation of ether reduces the energy of the cluster, which makes the formation of ether favorable. We then demonstrate that photonic gasification is a convenient tool for regulating the pore size. This allows for gasification to occur at room temperature, which is advantageous compared to thermal gasification. This is because, for the latter, clusters tend to grow in size by the coalescence of diffusing smaller clusters at elevated temperatures.^38^

We further demonstrate that the gasification of clusters by light at room temperature avoids cluster coalescence events and results in highly selective pores. Cluster density could be increased without concomitant gasification of the clusters by increasing the oxidation temperature to 80 °C. We show that gas permeance could be systematically increased while maintaining or even increasing the gas pair selectivity. We attribute this to the selective gasification of the strained cluster core. We further show that the Å-scale pores prepared in this way could selectively differentiate CH_4_ from N_2_, which is considered to be a challenging separation. Dedicated molecular dynamics simulations reveal a relatively stronger adsorption of CH_4_, as well as the relaxation of the C–H bond, inside the pores.

## Results

### Formation of Ether at the Core of the Epoxy Cluster

**Figure 1:**
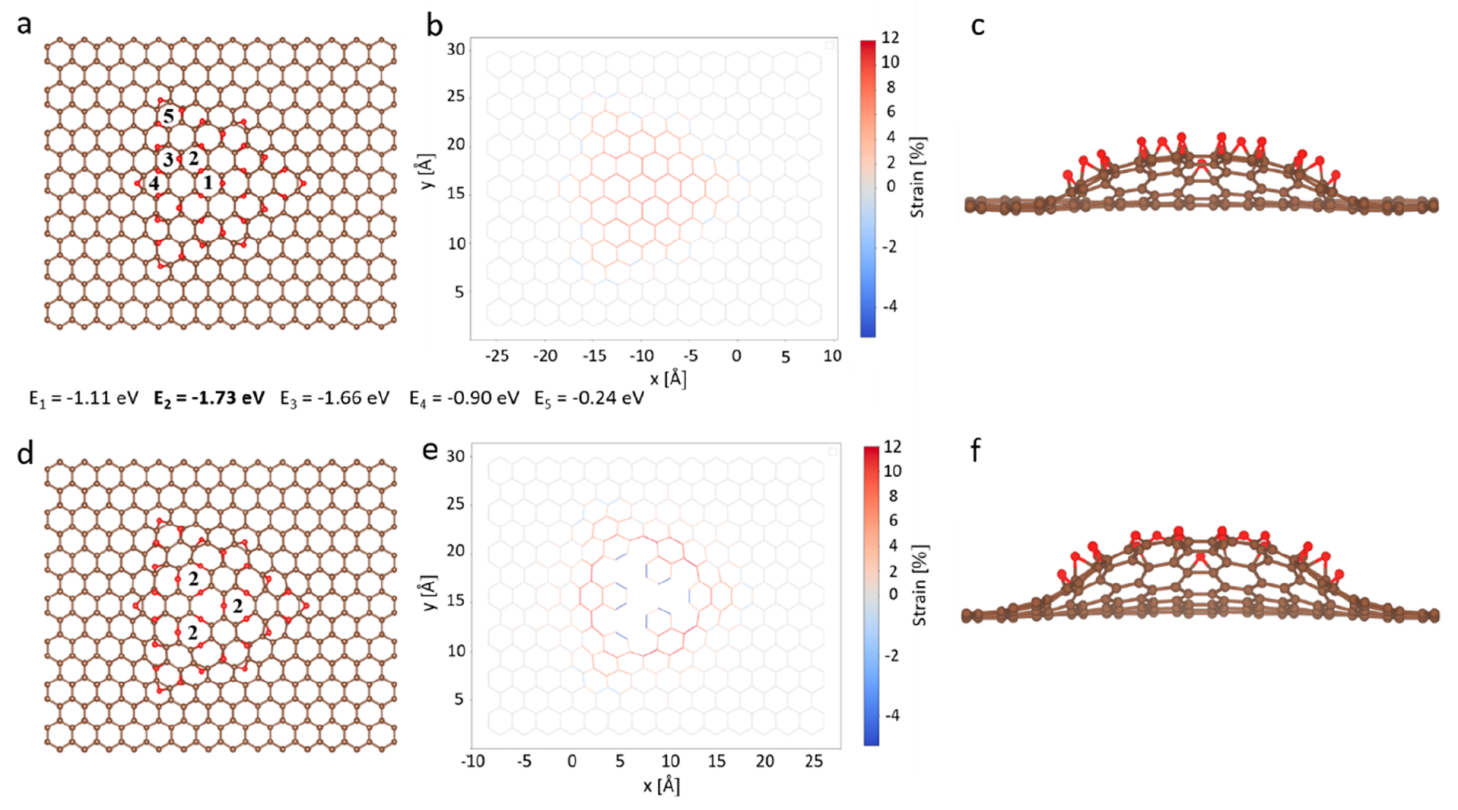
Prediction of evolution of ether groups inside epoxy cluster. (a) Structure of a 33 O atom epoxy cluster, where symmetrically nonequivalent trimer positions are marked as 1–5. (b) Map of C–C bond strain for the cluster in (a) with color-coded strain (blue signifies shorter C–C bond lengths and red signifies longer C–C bond lengths compared to pristine graphene). (c) Side view of the epoxy cluster depicted in (a). (d) Structure of a regular 33 O atom cluster displaying an ether core that consists of nine O atoms. The ether trimers positions are marked with “2”. (e) Map of C–C bond strain for the cluster depicted in (d). (f) Side view of the oxygen cluster depicted in (d).

Having an understanding of the structure, composition, and bond strain of the cluster is highly desired for performing selective manipulation via gasification in order to finely control the size of the resulting pore. When samples were oxidized at a temperature >25 °C, we observed a significant presence of ether and epoxy in the resulting samples (the experimental data is shown in the next section). Thus, we explored the formation of ether groups in the cluster. For this, we calculated the energetics of various epoxy and ether configurations with density functional theory (DFT). For the model systems, we considered an epoxy cluster of 33 O atoms (with a cluster diameter of 1.7 nm) grouped in a trimer configuration (three O atoms in a single honeycomb), which is the most preferred configuration for cluster organization (Figure 1a).^39^ Because of the radial symmetry of the cluster, there are only five possible sites for substituting epoxy trimers with ether trimers in this cluster. These are marked with the numbers 1–5 at progressively increasing distances from the core of the cluster (Figure 1a).

Setting the total energy of the cluster, *E*, to 0 eV (as reference) when it is made of only epoxy (as in Figure 1a), we discovered that the cluster energy decreases by a significant amount in all cases when an ether trimer replaces an epoxy trimer. This confirms that the formation of ether within the epoxy cluster is energetically favorable. We further discovered that the most favorable position for an ether trimer is close to the core of the cluster (position 2, *E* = −1.73 eV), followed by positions 3 (*E* = −1.66 eV), 1 (*E* = −1.11 eV), and 4 (*E* = −0.90 eV). The least favorable position for ether trimer formation is at the edge of the cluster (position 5, *E* = −0.24 eV). We also observed a similar trend for a smaller cluster made up of 24 O atoms (with a cluster diameter of 1.3 nm, Figure S1).

These results can be explained by the system’s tendency to reduce the strain in the C–C bond. When the cluster is solely made of epoxy groups, the largest strain on the C–C bond is at and around the core of the cluster (Figure 1b). The maximum bond length at the core of the cluster reaches ∼1.56 Å, as opposed to 1.42 Å in a pristine graphene lattice, thus corresponding to a large strain close to ∼10%. Here, percentage strain is defined as 100 × (bond length – 1.42)/1.42. Furthermore, we observed that the C–C bond strain is largely independent of the cluster size. To treat clusters of up to 159 O atoms (with a cluster diameter of 3.5 nm), we employed density-functional tight-binding calculations and confirmed that even in larger clusters, the C–C bond in the presence of an epoxy group is highly strained, reaching a maximum bond length of ∼1.56 Å (Figure S2). The large bond strain and accompanying large system energy are expected to relax the system by splitting of the C–C bond of the epoxy, resulting in the generation of ether.

We further explore the formation of a larger ether core inside of an epoxy cluster by switching more than one epoxy trimer to an ether trimer. For instance, we present a triple ether trimer configuration that can be considered to consist of the three most energetically favorable trimers at three symmetric locations for position 2 (Figure 1d,e). This results in a lower cluster energy (*E* = −3.74 eV) with respect to the epoxy cluster. This configuration is essentially a core/shell structure with epoxy at the shell and ether at the core. In this configuration, the expansion created by the C–O–C bond results in a large out-of-plane bulging of the cluster (Figure 1f), unlike what occurs in the epoxy cluster (Figure 1c).

### Selective Photonic Gasification

**Figure 2:**
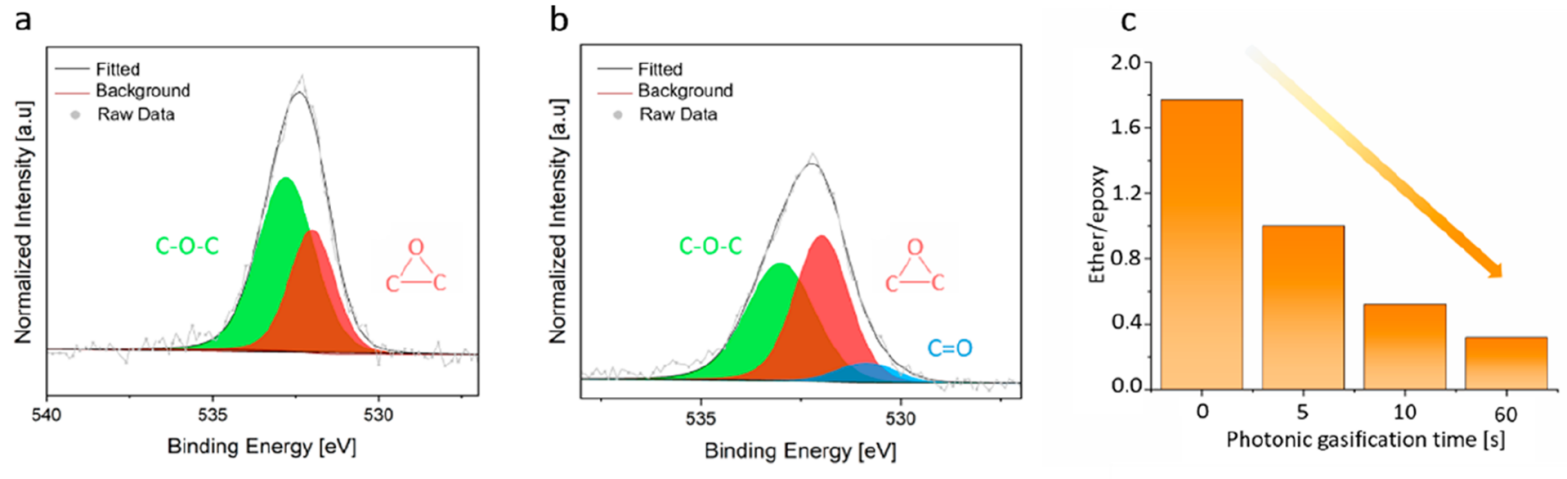
Selective photonic gasification of oxygen clusters on HOPG oxidized at 80 °C for 1 h. (a) The normalized O1s XPS spectrum of the oxidized HOPG. (b) The O1s XPS spectrum of the oxidized HOPG lattice after 5 s of exposure to 3.2 eV of light. (c) Corresponding ether/epoxy population and its evolution as a function of gasification time.

Gasification by light irradiation is attractive because (i) it can be done at room temperature, (ii) it limits cluster diffusion and coalescence events, (iii) one can dose a precise amount of energy, and (iv) the gasification time is very small (only a few seconds compared to 1 h for thermal gasification). To understand photonic gasification, highly oriented pyrolytic graphite (HOPG) was oxidized in a homemade oxidation setup. This involved exposing HOPG to O_3_ for 1 h.^37^ The O1s peak from the high-resolution X-ray photoelectron spectroscopy (XPS) data were analyzed to understand the composition of the O functional groups formed by oxidation (Figure 2a). When oxidation was carried out at 80 °C, the O functional groups were made of ether (64%) and epoxy (36%). This contrasts with low-temperature oxidation (25 °C) results, where epoxy is dominant (91%) and ether is only present as a minor component (9%).^37^ This difference is due to the fact that, when oxidation is carried out at an elevated temperature, it increases the population of large clusters via enhanced epoxy diffusion, which has a low energy barrier (0.7 eV).^37^ An increase in the size of the O cluster is also reported for graphene oxide upon thermal annealing.^38^ Large clusters develop lattice strain at the core, which in turn promotes the formation of ether, as discussed in the previous section.

We did not observe semiquinone groups in the as-oxidized sample with a maximum oxidation temperature of 80 °C. This provides evidence of the absence of pores in the as-oxidized sample, as semiquinone groups can only be present in the presence of vacancy defects given the bonding requirement of C (which will be discussed later).^37,40^ Another indication of oxidation came from the Raman data of oxidized single-layer graphene on Si/SiO_2_, which appeared similar to that for GO, marked by broad D and G peaks and a negligible 2D peak (Figure S6).^41^

Next, we carried out room-temperature photonic gasification by exposing the as-oxidized sample to 3.2 eV of light (390 nm, 4 W cm^–2^, 0.5 cm from the sample) for a short time (5 s, Figures S3 and S4). A significant difference in the composition of the cluster before and after light exposure was observed (Figure 2b). In particular, the ether population decreased significantly compared to that of epoxy. The relative population of ether with respect to epoxy continued to decline with progressive exposure to light (Figures 2c and S5). We attribute this to the gasification of the strained cluster core primarily occupying ethers. Semiquinone functional groups were generated after light exposure, which provides evidence of pore generation (Figure 2b).^36^ Given that not all of the O functional groups were gasified during the 5 s of exposure, donut-shaped pores should have resulted where vacancy defects were surrounded by the remaining ether/epoxy groups (Scheme 1), as reported recently by scanning tunneling microscopy.^40^

Although pristine single-layer graphene is a zero-bandgap material, introducing the presence of an oxygen cluster breaks the system’s symmetry, distorts the lattice, and introduces a bandgap at the functionalized site.^42^ For example, an O-coverage-dependent bandgap of up to 3.0 eV has been reported for graphene oxide,^43,44^ which is well within the excitation reach of the 3.2 eV of light used in this study. This explains why photonic gasification is effective for an oxygen-functionalized graphitic lattice.

### Independent Control of Pore Density and Size

Photonic gasification at room temperature restricts cluster migration and therefore cluster–cluster coalescence, which otherwise promotes the formation of larger pores. This allowed us to probe whether cluster (pore) density could be increased by increasing the extent of oxidation while simultaneously maintaining a narrow pore size. For this, the oxidation temperature was varied from 43 to 80 °C. We limited the temperature to 80 °C because thermal gasification takes place above 80 °C (e.g., 100 °C).^37^ Thus, by oxidizing at or below 80 °C, we avoided pore formation, decoupling cluster formation, and pore formation processes. In all cases, the oxidation time was 1 h, and photonic gasification was carried out by 5 s of exposure to 3.2 eV of light. Following this, the resulting porous graphene was suspended on a macroporous support by mechanical reinforcement using a support film, following a procedure reported previously, in order to carry out a gas transport study (see Materials and Methods for details).^29,45^

**Figure 3:**
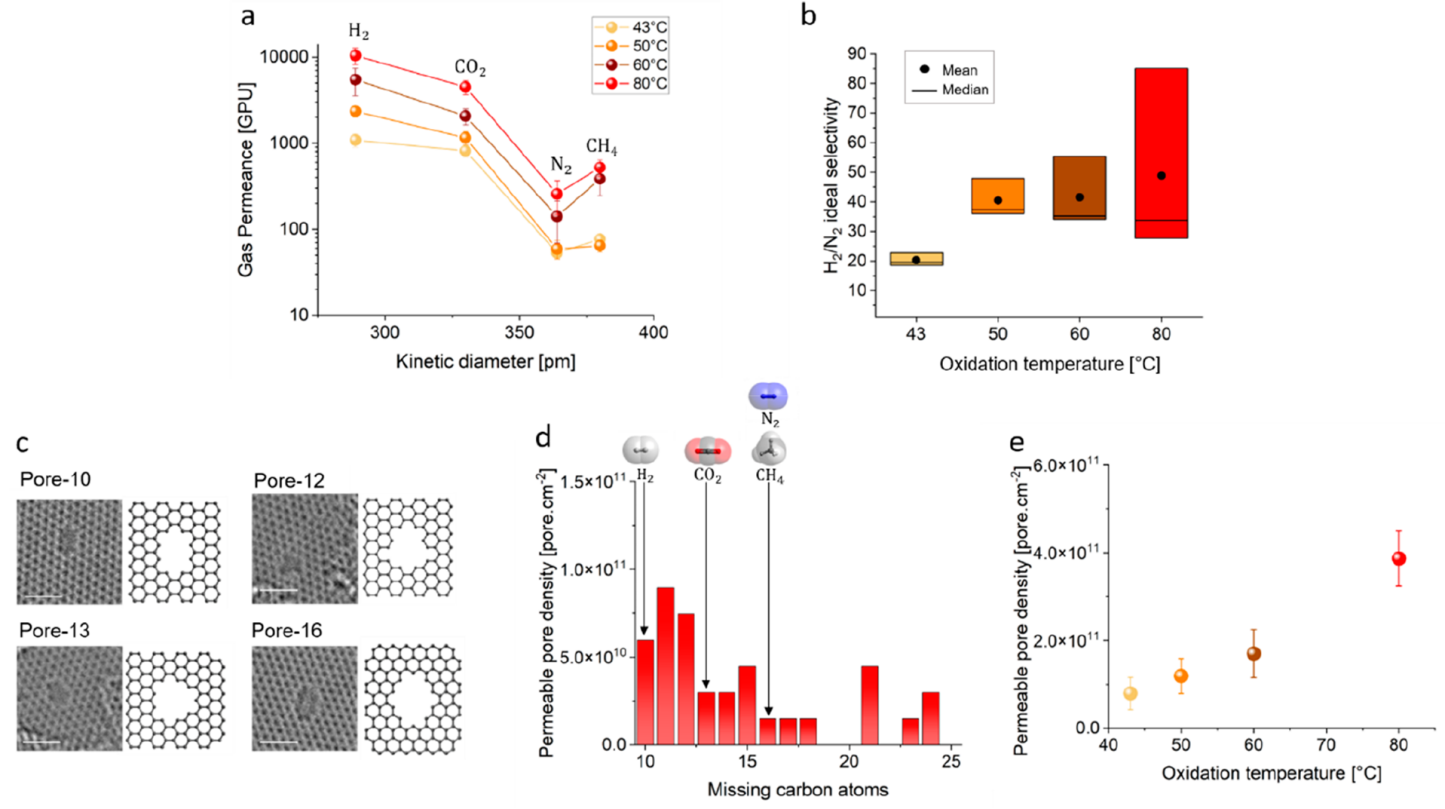
Photonic gasification to overcome the trade-off between gas permeance and ideal gas selectivity. (a) Gas permeance evolution as a function of the oxidation temperature. The permeance data were collected at 30 °C. (b) H_2_/N_2_ pair selectivity as a function of oxidation temperature. The standard deviation is from the data from three samples. (c) AC-HRTEM images of H_2_-permeable pores generated at an oxidation temperature of 80 °C and with 5 s of light gasification. Scale bar: 1 nm. (d) Size distribution of H_2_-permeable pores in terms of number of missing carbon atoms obtained from the AC-HRTEM data (see Supplementary Note S2 (p 9 of the Supporting Information) for details). (e) Trend in the density of H_2_-permeable pores as a function of the oxidation temperature. The error bars represent the standard deviation in mean from at least eight AC-HRTEM images.

The first evidence of the formation of Å-scale pores from the photonic gasification at room temperature came from the trend in the permeance of small gas molecules. The gas permeance of H_2_ was much higher than that from the intrinsic defects in the as-synthesized graphene, indicating an increased porosity of the graphene (Table S1). Also, the permeance decreased for larger gas molecules in the following sequence: H_2_ > CO_2_ > N_2_. This result confirms that pore-differentiated gas molecules are based on their size rather than their mass. Therefore, the size of the gas molecule with respect to the pores regulated the gas transport (Figure 3a). This is further confirmed by the high H_2_/N_2_ selectivity of all of the samples (in the range of 20–85, Figure 3b), which is well beyond that of the reinforcing layer (∼6.7, Table S2).

To regulate the pore density, we controlled the oxidation kinetics by varying the reaction temperature. The reaction kinetics are promoted at higher temperatures because higher temperatures help to overcome the energy barrier for the chemisorption of ozone (0.72 eV).^46^ High temperatures also help to overcome the cluster nucleation energy barrier.^37^ Indeed, we observed that the gas permeance monotonically increased with the increase in the oxidation temperature (43, 50, 60, and 80 °C; see Figure 3a).

Generally, one always observes a trade-off between gas permeance and gas pair selectivity because the conventional routes for pore incorporation have concomitant pore formation and expansion, which leads to increased pore size at a higher pore density.^35,36^ However, in the approach we employed, we were able to successfully increase both gas permeance and gas pair selectivity in a systematic way. For the smallest gas that we probed (H_2_), the increase in gas permeance was significant (a 12-fold increase between 43 and 80 °C). The increase was relatively less for larger gas molecules (e.g., N_2_ had a 4-fold increase). This indicates that the density of the Å-scale pores selective to H_2_ increased as a function of the oxidation temperature. We attribute this to the unique aspects of this study: site-specific pore generation through decoupled cluster formation and gasification at room temperature with the use of light. The latter prevents cluster coalescence by epoxy diffusion, which is inevitable in thermal gasification.^37^ To further probe this, we carried out a control experiment where gasification of graphene oxidized at 80 °C was carried out by heating at 500 °C instead of with room-temperature photonic gasification (Figure S7). While we also observed selective transport in this experiment, the obtained H_2_/N_2_ selectivity was much smaller (∼13) compared to that yielded by photonic gasification. Thus, it is clear that photonic gasification allowed us to realize an attractive combination of H_2_ permeance (∼12 000 gas permeation units (GPU); 1 GPU = 3.35 × 10^–10^ mol m^–2^ s^–1^ Pa^–1^) and H_2_/N_2_ selectivity (with an average selectivity of 48.8).

To confirm that pore density increased as a function of the oxidation temperature, we carried out imaging of the porous graphene lattice at atomic resolution. For this, AC-HRTEM at 80 kV was carried out with a low beam dose (1.0 × 10^4^ e^–^ Å^–2^ s^–1^). Under these imaging conditions, pore expansion was not observed (using a maximum beam exposure time of 10 s); however, pore-edge functionalization was lost because of the electron beam energy.^37^ Given that pore-edge carbon atoms likely gasify with the functional group, the pore size distribution observed by AC-HRTEM is a slight overestimation. However, the distribution data are very useful for understanding pore density and trends in size distribution.

We indeed observed an increasing density of vacancy defects as the epoxidation temperature increased (2.6 × 10^12^ and 5.5 × 10^12^ cm^–2^ at 43 and 80 °C, respectively; see Figure S9). This population of vacancy defects included all defects, including small defects that were not expected to permeate gases. To understand the trend for gas-permeable defects or pores, we screened O-functionalized pores using their van der Waals (vdW) gap to understand the permeation probability for gas molecules for a set of pores (Supplementary Note S2 and Table S4).^18^ This analysis showed that H_2_ would not permeate through small pores such as O-functionalized pore-6 or pore-8 (where 6 and 8 denote the number of missing carbon atoms); however, H_2_ could permeate through O-functionalized pore-10. CO_2_ would not permeate through pores smaller than the O-functionalized pore-13. CH_4_ and N_2_ would not permeate through pores smaller than the O-functionalized pore-16. Therefore, the incorporation of pores with sizes between pore-10 and pore-16 is attractive for the selective transport of H_2_ and CO_2_.

The pore size distribution of the sample prepared by oxidation at 80 °C revealed that the majority of the pores were indeed smaller than pore-16 (Figure 3d). This explains the observed high H_2_/N_2_ selectivity. In other words, H_2_ permeated the fastest because it had access to a higher density of permeable pores compared to the larger gas molecules. We extracted the density of H_2_-permeable pores from AC-HRTEM data for the samples oxidized at 43, 50, 60, and 80 °C (Figure 3e). These results indeed confirm that the density of H_2_-permeable pores increased significantly from 43 to 80 °C. On the basis of the pore density at 80 °C, we estimated the permeability coefficient of H_2_ as 10^–21^ mol s^–1^ Pa^–1^, which is consistent with the literature on H_2_ transport through Å-scale graphene pores, where H_2_ experiences an energy barrier to cross the pores.^34,47^ Indeed, the temperature-dependent H_2_ transport revealed a small energy barrier of 11 kJ mol^–1^.

### Separation of CH_4_ from N_2_

**Figure 4:**
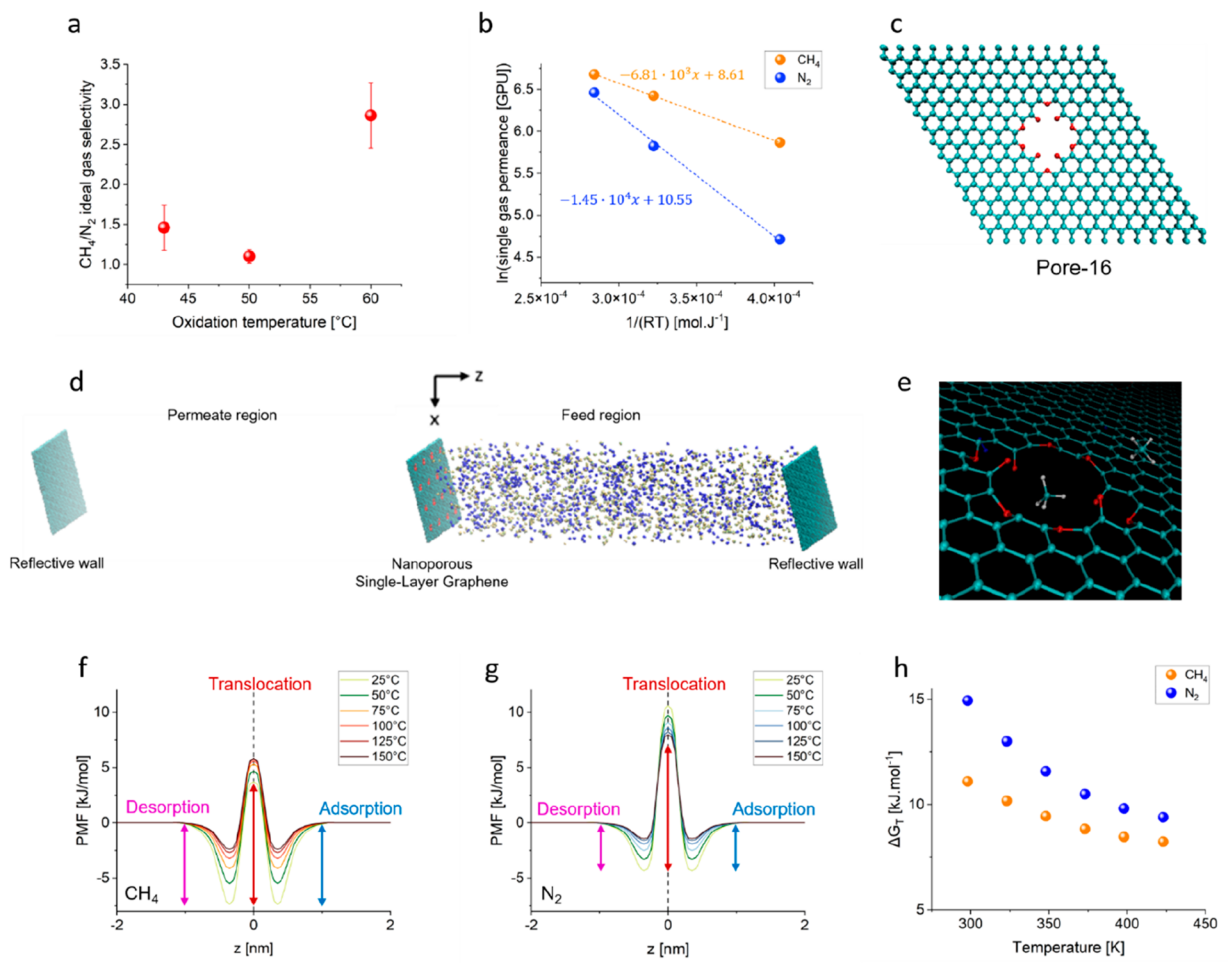
Investigation of the permeation of CH_4_ and N_2_ across the graphene pores. (a) CH_4_/N_2_ ideal gas selectivity for various O_3_ functionalization temperatures, followed by 5 s of light gasification. The standard deviation is from the data from three different samples. (b) Arrhenius fitting of ideal gas permeance for both CH_4_ and N_2_ for pores generated at an O_3_ functionalization of 60 °C with 5 s of light gasification. (c) Pore-16, which has 16 carbon atoms removed and its pore edge terminated by ether and semiquinone. (d) The MD configuration that was used to study the permeation of CH_4_ and N_2_ through the graphene pores. (e) Snapshot of CH_4_ permeating across pore-16. (f) Potential of mean force (PMF) profiles for CH_4_ permeation across pore-16 at various temperatures. (g) PMF profiles for N_2_ permeation across pore-16 at various temperatures. (h) Free energy barrier for pore translocation (Δ*G*_T_) for CH_4_ and N_2_ as a function of temperature.

Separating CH_4_ from N_2_ is challenging because their kinetic diameters are quite similar (3.8 and 3.64 Å, respectively).^48−52^ This separation is attractive for natural gas purification when N_2_ is mixed in CH_4_, and it may become critical for reducing the emission of CH_4_, a potent greenhouse gas, into the atmosphere.^53^ Given that the obtained pore size distribution indicated the presence of CH_4_- and N_2_-permeable pores (pores that are equal to or larger than pore-16; see Figure 3), we sought to understand whether the pores could differentiate this gas pair. Interestingly, CH_4_ permeated faster than N_2_, as the CH_4_ permeance and CH_4_/N_2_ selectivity somewhat increased with the oxidation temperature (Figures 4a and S25). At an oxidation temperature of 60 °C, an attractive combination of CH_4_ permeance (∼400 GPU) and CH_4_/N_2_ selectivity (∼3) was achieved (Figures 4a and S25). These results are surprising, as N_2_ is a smaller molecule, especially when it is oriented vertically when crossing a pore.^54^ Nevertheless, the mechanism behind such an unusual permeation is not clear.^55,56^ To understand this further, we measured the temperature-dependent transport of CH_4_ and N_2_ and observed that their transport is thermally activated (Figure 4b).^45,55,56^ Extracting the energy barrier from the activated transport model yielded a much higher energy barrier for N_2_ (14.5 kJ mol^–1^) compared to CH_4_ (6.8 kJ mol^–1^).

To gain mechanistic insight for this separation, molecular dynamics (MD) simulations for the permeation of CH_4_ and N_2_ across the graphene pores were carried out. Given that vdW screening identified the O-functionalized pore-16 to be permeable for both CH_4_ and N_2_, we used this pore for the MD simulations (Figure 4c). In the pursuit of being as close as possible to the experimental conditions, a low feed pressure of 10 bar was used. An equimolar feed was used, while a vacuum was maintained on the permeate side (Figure 4d).

Potential of mean force (PMF) profiles along the *z*-axis for both CH_4_ and N_2_ permeating across pore-16 were obtained using the umbrella sampling method.^57,58^ Three different regions corresponding to the three subsequent permeation steps (adsorption, translocation, and desorption) were identified from the PMF profiles of the two gases (Figure 4f,g).^56^ The binding energy, extracted from the PMF valley, predicted a stronger affinity of CH_4_ (7.4–2.4 kJ mol^–1^) to the graphene pores compared to N_2_ (4.4–1.4 kJ mol^–1^) in the temperature range of 25–150 °C. This result is consistent with the higher condensability of CH_4_ compared to N_2_.^59,60^ Consistent with previous studies, for both gases, the rate-limiting step for permeation was identified as the pore translocation step. Therefore, the free energy barrier for translocation (Δ*G*_T_) was computed as a function of the permeation temperature (25–150 °C, Figure 4h). Briefly, Δ*G*_T_ was calculated based on the difference in PMF between the pore mouth (the transition state for translocation) and the valley (the adsorbed state).^56^ Interestingly, at all temperatures, the Δ*G*_T_ values for CH_4_ were found to be lower than those of N_2_, which is consistent with the higher permeation rates of CH_4_. Especially at 25 °C, Δ*G*_T_ for CH_4_ (11.1 kJ mol^–1^) was 2.6*k*_B_*T* lower than that for N_2_ (14.9 kJ mol^–1^), thus resulting in CH_4_ permeating more rapidly compared to N_2_, as was observed in the experiments (Figure 4b,h). With an increase in temperature, the Δ*G*_T_ values for both N_2_ and CH_4_ decreased, resulting in higher permeation rates at elevated temperatures (Figure 4h). We also observed that Δ*G*_T_ as a function of temperature follows a linear trend (*R*^2^ > 0.94), and therefore, the enthalpic and entropic contributions to the gas transport could be obtained (Figure S28). The lower enthalpic barrier for CH_4_ transport (18.4 kJ mol^–1^) compared to that for N_2_ transport (27.4 kJ mol^–1^) is consistent with the faster permeation of CH_4_ through the pores (Figure S29).

## Conclusion

Overall, in this study we demonstrate that cluster (pore) density can be independently modulated by the oxidation temperature. We gasified clusters by exposing them to 3.2 eV of light at room temperature to achieve Å-scale pores. These pores formed by the gasification of the cluster core, which is evident by the decrease in the ether/epoxy ratio. Photonic gasification at room temperature led to an attractive gas separation performance, in which both gas permeance and gas selectivity could be increased simultaneously. We show that manipulating the pore precursor (i.e., the cluster) at room temperature is an effective way to control the generated pores with high precision, as required for achieving challenging molecular separations (e.g., separating CH_4_ from N_2_). This also opens a new direction in further manipulating the cluster, e.g., by changing the irradiation energy and exposure time, by converting epoxy and ether into other chemical species via functionalization, etc. Finally, the use of light to form pores at room temperature is convenient and will accelerate efforts to scale up the application of porous single-layer graphene.

## Materials and Methods

### Annealing of the Commercial Catalytic Foil and Growth of Single-Layer Graphene

Growth of the single-layer graphene film was carried out on a catalytic Cu foil by chemical vapor deposition (CVD), as reported previously.^45^

### Photonic Gasification

Graphene resting on copper was exposed to ozone at various temperatures in a homemade setup using 13.4 wt % O_3_ in O_2_ for 1 h.^37^ Subsequently, the oxidized graphene was exposed directly to light (3.2 eV) at room temperature for 5 s at a distance of 0.5 cm from the light source for a maximized radiation dose (Phoseon, Firefly 25x10AC395-4W).

### Membrane Fabrication

The sample that was prepared by photonic gasification was reinforced by a nanoporous carbon (NPC) film that was reported in previous work. A second mechanical reinforcement made of a 250 nm thick poly[1-(trimethylsilyl)-1-propyne (PTMSP) film was applied.^29,33^ Next, copper was etched away in a 0.5 M iron chloride solution, and the sample was further washed in 1 M HCl and water baths. Finally, the film was transferred onto a macroporous W foil support hosting 5 μm holes.^45^

### Gas Permeation Measurement

The single gas components were measured using a homemade permeation module (Figure S30). All of the units used in the setup were calibrated within a 5% error margin, including the mass flow controllers, mass spectrometer (MS), and oven. The porous graphene films resting on W foil were sandwiched directly in a Swagelok VCR-based module in order to ensure a leak-proof seal. In order to avoid any temperature fluctuations, the sweep and feed lines were preheated directly in the oven. The feed pressure was controlled by using a back-pressure regulator. To ensure accurate gas permeation measurements, the MS was calibrated for all of the gases investigated in this study. To remove any contaminants present on the membrane surface, all membranes were heated inside the gas module to 150 °C for 1 h before recording the gas permeance.^45^

### XPS

The XPS investigations of HOPG were completed with an Axis Supra instrument (Kratos Analytical) by using the monochromated Kα X-ray line of an aluminum anode. The step size was set to 0.1 eV, and the pass energy was set to 40 eV. To avoid charge buildup upon measuring, the samples were grounded. To analyze the data, the CasaXPS software was used, and the Shirley method was applied to subtract the background. Lastly, the collected binding energy data were used without any further correction.

### Freestanding Graphene for AC-HRTEM

Following CVD, the graphene film was transferred from the copper foil to a PELCO silicon nitride grid with 1 μm holes using the paraffin-based transfer method.^37^ The resulting suspended graphene on the grid was oxidized in an ozone flow for 1 h, and the pores were exposed via photonic gasification following the same approach as for the fabrication of the membranes.

### AC-HRTEM Imaging and Analysis

Imaging of the graphene pores was carried out with a Thermo Fisher Scientific Titan Themis microscope using an electron beam with an acceleration voltage of 80 kV. The TEM was equipped with a 4K-resolution complementary metal-oxide semiconductor (CMOS) camera (Thermo Fisher Scientific, Ceta) and an image aberration corrector (CEOS, CETCOR), along with a monochromated high-brightness field emission gun (X-FEG).

In the pursuit of minimizing chromatic aberrations, the incident electron beam was monochromated. To maximize the image resolution, geometric aberrations were corrected up to the fourth order with a negative Cs of ∼18 μm. Videos were recorded at a magnification of 520 000 at a maximum dose rate of 2 × 10^4^ e^–^ Å^–2^ s^–1^, an exposure time of 0.2 s, and a camera binning of 4. Images were obtained by integrating 5–15 consecutive images from the recorded videos (each image that was considered for the integration was taken with an exact acquisition time of about 200 ms). To enhance the image quality, a bandpass filter was used, enhancing the lattice resolution.

The number of missing carbon atoms for vacancy defects were quantified using a graphical approach that has been reported in a previous work (Figure S8).^33^ Briefly, the pore contour was drawn manually before being superposed onto a graphene lattice, scaled to the right magnification (520 000). Subsequently, the carbon atoms that were covered by the vacancy were deleted. Finally, using the ImageJ software, the number of missing carbon atoms were quantified. Acknowledging the potential mismatch between the graphene lattice and the TEM image, a correction factor, which depended on the length of the contour line, and geometric factors (the thickness of the contour line for the pore edge, the size of the carbon dots compared to the estimated lattice, and the degree of mismatch between the contour and digital lattice) were used to correct the number of missing atoms.

### Density Functional Theory Calculations for Cluster Morphology Estimation

First-principles calculations were performed at the density functional theory level using the semilocal Perdew–Burke–Ernzerhof (PBE) functional, as implemented in the Vienna Ab initio Simulation Package (VASP) code.^61,62^ Kohn–Sham wave functions were expanded in a plane-wave basis set with a kinetic energy cutoff of 400 eV, while electron–core interactions were described through the projector augmented wave (PAW) method.^63,64^ We adopt the Grimme DFT-D3 approach to describe the van der Waals interactions.^65^ A conjugate gradient method was used to optimize the atomic positions and lattice constants, where the total energy and atomic forces were minimized. Convergence was reached when the maximum force acting on each atom was <0.02 eV Å^–1^, while the next energy step was <10^–6^ eV. For the epoxy cluster calculations, a 15 × 15 graphene supercell was used with a 3 × 3 × 1 *k*-point mesh grid to sample the Brillouin zone, while an epoxy-covered graphene unit cell was sampled with a 9 × 9 × 1 *k*-point mesh grid. All structures were subjected to periodic boundary conditions with a vacuum layer of 10 Å in the direction perpendicular to the layers in order to prevent interactions between replica images.

### Density Functional Theory Calculations for the Optimal Relaxed Pore Structure

The Quantum ESPRESSO software was used to obtain the optimal relaxed structure of porous graphene.^66−68^ Plane-wave basis sets were used, and for the electronic wave function expansion, cutoffs of 50 and 500 Ry were employed for the wave function and charge density, respectively. Exchange correlations were described using the PBE functional.^61^ Ultrasoft pseudopotentials were employed for the interactions between the ionic core and the valence electrons.^69^ A vacuum of 20 Å was used to avoid interactions between the periodic images along the direction normal to the functionalized porous graphene surface. Because of the large supercell, Brillouin zone sampling was restricted to the Γ point. The Broyden–Fletcher–Goldfarb–Shanno scheme was employed to perform structural relaxation until the Hellmann–Feynman forces were <0.001 Ry bohr^–1^. London dispersion corrections were described using the DFT-D2 functional.^70^ The relaxed unit cells of the functionalized porous graphene were replicated in the *x*–*y* plane to generate functionalized porous graphene with 16 functionalized pores.^16,71^

### Molecular Dynamics Simulations

Molecular dynamics simulations were performed using the GROMACS 5.1.4 simulation package.^72,73^ All atoms of the functionalized porous graphene except for the functional groups terminating the pores were fixed in their respective atomic positions throughout the simulation.^16^ For all cases, the reflective walls and functionalized porous graphene were located perpendicular to the *z*-axis. Owing to the hexagonal lattice of graphene, a parallelepiped simulation box was used.^16,71^

For the gas permeation studies, a gas reservoir with 1000 N_2_ molecules and 1000 CH_4_ molecules was first equilibrated at temperatures of 25, 50, 75, 100, 125, and 150 °C in an NVT ensemble. The equilibrated gas reservoir was then enclosed by the functionalized porous graphene and a reflective wall. On the permeate side, another reflective wall was placed at the same distance from the functionalized porous graphene as the reflective wall on the feed side. Finally, a vacuum of 5 nm was extended beyond the reflective walls to ensure sufficient space between the periodic images of the system along the *z*-direction. Periodic boundary conditions were applied in all three directions.

All-atom optimized potentials for liquid simulation parameters were used for the functionalized porous graphene, CH_4_, and N_2_.^19,22,56,74^ Nonbonded interactions were modeled using dispersive and electrostatic forces. Van der Waals interactions were modeled using Lennard-Jones potential with a cutoff of 1.2 nm. The Lorentz–Berthelot mixing rules were applied for the cross-interaction parameters for the Lennard-Jones potential between other unlike pairs. The particle mesh Ewald (PME) algorithm was used to compute the long-range electrostatic interactions with a cutoff of 1.2 nm for real-space force calculations.^75^ The leap frog algorithm was employed to integrate the equations of motion with a time step of 1 fs. The temperatures were maintained using the V-rescale thermostat with a time constant of 0.2 ps.^76^ Three sets of production runs of 10.5 ns each were performed, where the first 0.5 ns was treated as the equilibration stage.

### Potential of Mean Force Calculations

The umbrella sampling method was used to calculate the free energy barrier for gas permeation through the functionalized graphene pores at different temperatures.^57,58^ Force constants of 500–1000 kJ mol^–1^ nm^–2^ were used in the harmonic umbrella potential, and the *z*-coordinate of the probe gas molecule was varied from 2.0 to −2.0 nm in decrements of 0.05 nm, with the functionalized porous graphene located at *z* = 0. This resulted in 81 windows along the *z*-direction. Each window was sampled for at least 6 ns, where the first 0.5 ns was treated as the equilibration stage. The collected data were analyzed using a weighted histogram analysis method.^77^

## Supporting Information Available

Supplementary notes, DFT analysis, XPS data, HRTEM images and analysis, permeable pore screening analysis, MD analysis, gas permeation data, and information on the experimental setups (PDF)
