# Supplementary material for: Selective Photonic Gasification of Strained Oxygen Clusters on Graphene for Tuning Pore Size in the Å Regime
Source: JACS Au. 2023 Sep 29;3(10):2844–54. doi: 10.1021/jacsau.3c00395 (PMC10598578; doi:10.1021/jacsau.3c00395)
Supplement: Supplementary file 1 — au3c00395_si_001.pdf [file au3c00395_si_001.pdf]

Supplementary Information

Selective photonic gasification of strained oxygen clusters on graphene for tuning pore size in Å-regime

Luc Bondaz[1], Anshaj Ronghe[2], Shaoxian Li[1], Kristiāns Čerņevičs[3], Jian Hao[1], Oleg V. Yazyev[3], K. Ganapathy Ayappa[2], Kumar Varoon Agrawal[1,*]

1. Laboratory of Advanced Separations, Institute of Chemical Sciences & Engineering, École Polytechnique Fédérale de Lausanne (EPFL), CH-1950 Sion, Switzerland
2. Department of Chemical Engineering, Indian Institute of Science, Bangalore 560012, India
3. Institute of Physics, EPFL, Lausanne CH-1015, Switzerland

*Corresponding author: kumar.agrawal@epfl.ch

Supplementary Note S1

Investigation of ether formation location in 24 oxygen epoxy cluster by density functional theory (DFT)

Similar to oxygen cluster formed by 33 atoms, we investigated the energetically most favorable location of ether formation inside epoxy cluster formed by 24 O atoms. To do so, the epoxy cluster energy was set to 0.0 eV. Then, epoxy trimers at different locations were switched to ether trimers while examining the energy level of new configurations (Fig. S1). We obtain the energetics systematically by increasing distance from the core of the oxygen cluster (the higher the configuration number, the more distant the ether trimer is from the cluster core). Similarly, to 33 O cluster, the most favorable location for ether trimer formation, is at the core of the cluster (position 1, E = -1.046 eV), followed by 2 (E = 0.721 eV), 3 (E = -0.309) and 4 (E = +0.144 eV). Therefore, formation of ether trimer is energetically favorable in the core of the cluster while it is not favorable on the edge of the cluster. Such observation is greatly consistent with cluster formed by 33 oxygen atoms. Regarding the energy differences between the trimers at the edge (position 3 and 4), we note that position 4 is noticeably further away from the centre of the cluster with d_4= 5.36 Å, compared to position 3 (d_3= 4.65 Å). We also observe a higher C-C bond compression in the ether trimer, when it forms at position 4 (-5.58%) compared to position 3 (-5.19%), which makes trimer at position 4 more energetic than the trimer at position 3.

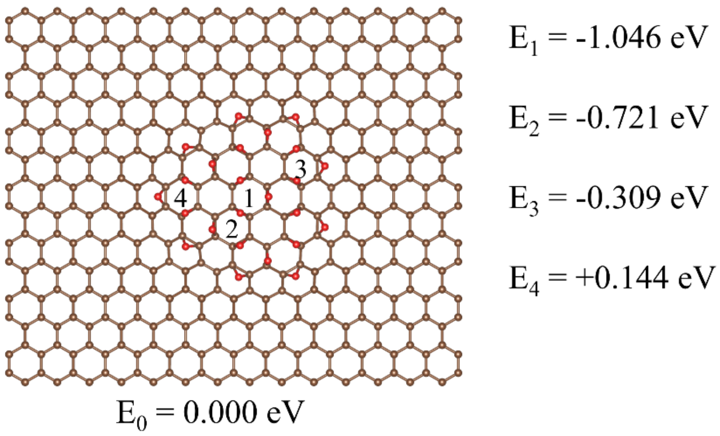

Figure S1. Prediction of oxygen cluster morphology formed by 24 oxygen atoms. Left, atomic structure of epoxy cluster formed by 24 oxygen atoms. The epoxy cluster is taken as a reference system and its energy is set to E = 0 as seen below its atomic structure. The numbers refer to potential locations of ether trimer formation, the higher the number the more distant the trimer would be from the cluster core. Right, energy with respect to epoxy cluster associated by the formation of ether at the different location depicted on left.

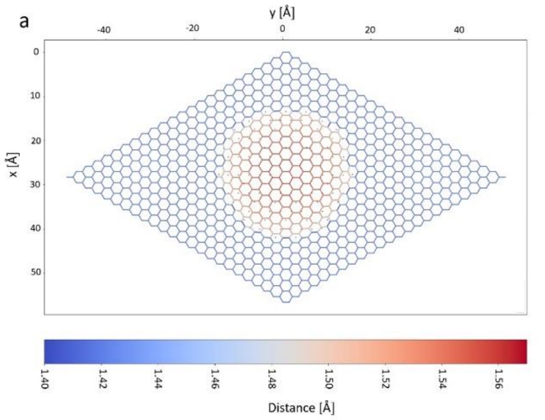

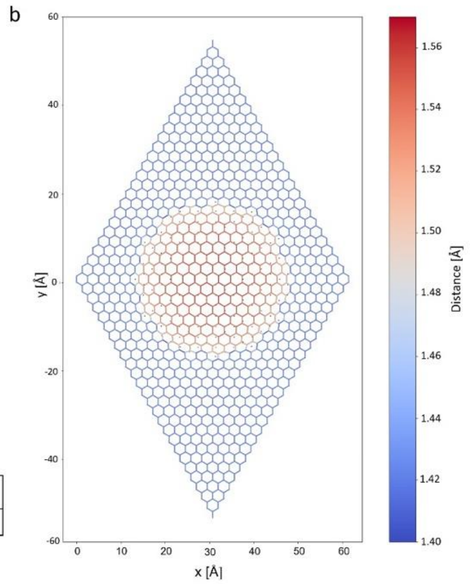

c

Oxygen atoms	24	33	45	87	118	159
Max bond length [Å]	1.556	1.562	1.561	1.555	1.555	1.557

Figure S2. Evolution of C-C bond length in the core of epoxy cluster as a function of the cluster size. a) Bond length map of the oxygen cluster formed by 118 oxygen atoms. Oxygen atoms are highlighted by blue dots. b) Bond length map of oxygen cluster formed by 159 oxygen atoms. Oxygen atoms are highlighted by blue dots. Such representations account for the 3D structure of the cluster where epoxy is out of plane. c) Maximum C-C bond length in epoxy clusters as a function of the cluster size.

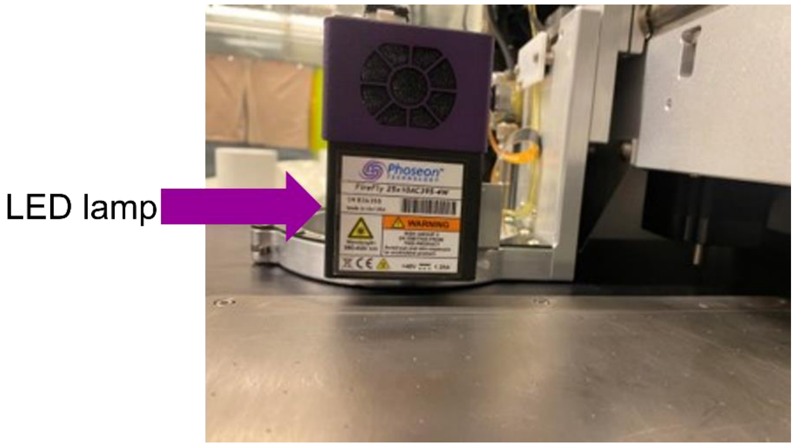

Figure S3. Photograph of the 3.2 eV lamp used for room temperature gasification.

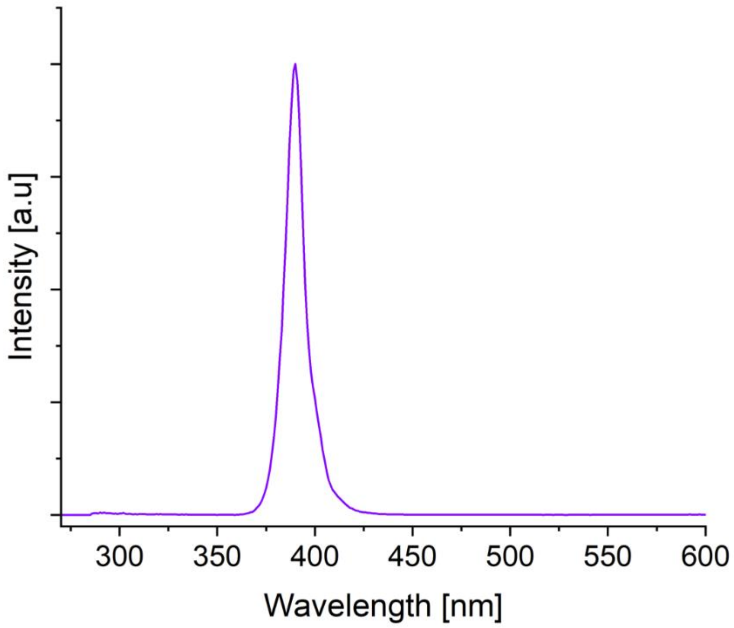

Figure S4. Wavelength distribution of the 3.2 eV lamp used in this work. The maximum intensity peak is located at 390 nm.

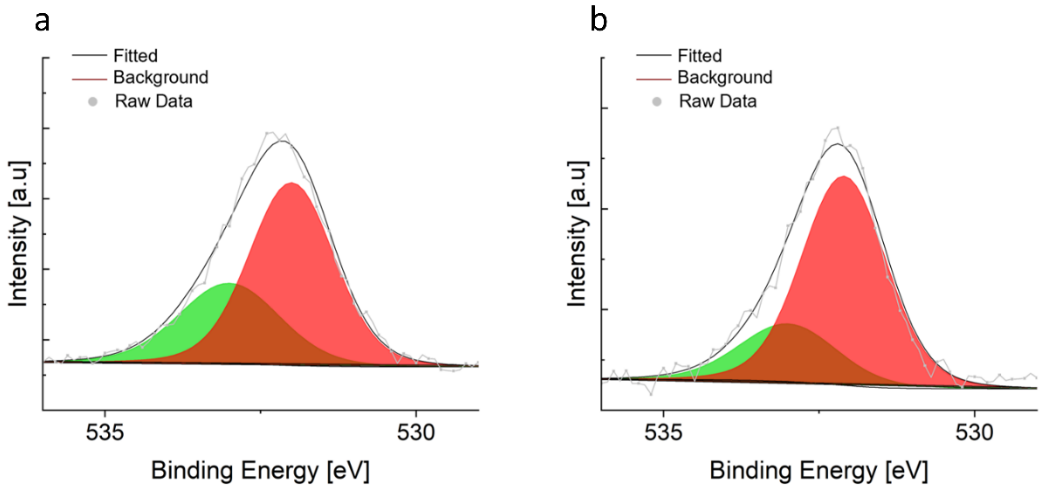

Figure S5. XPS data highlighting changes in the cluster composition after photonic gasification. O1s XPS spectrum of the oxidized HOPG lattice (in O_3 flow for 1 h at 80 °C) after exposure to 3.2 eV light for (a) 10 s and (b) 60 s. O_3 concentration was 13.4 wt% in O_2.

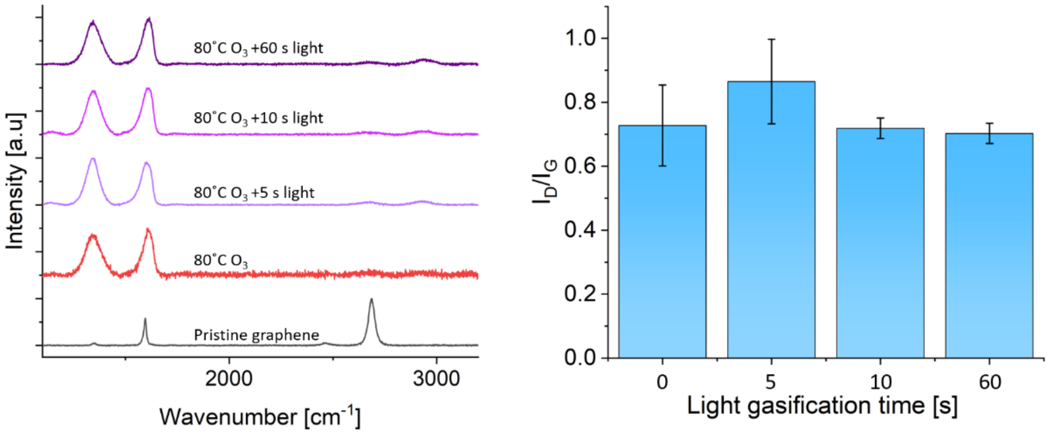

Figure S6. Raman data on oxidized graphene before and after gasification. Left: Raman spectroscopy for pristine, oxidized graphene and different photonic reduction time. Right: I_D/I_G evolution as a function of photonic gasification time.

Table S1. H_2 permeance through intrinsic defects in as-synthesized graphene.

	H_2 permeance [GPU]
Sample 1	346
Sample 2	133
Sample 3	138

Table S2. Gas permeance and ideal gas selectivity through the support film (NPC film reinforced by a PTMSP).

Gas	Permeance [GPU]
H_2	30251
N_2	4536

	Ideal gas selectivity
H_2/N_2	6.7

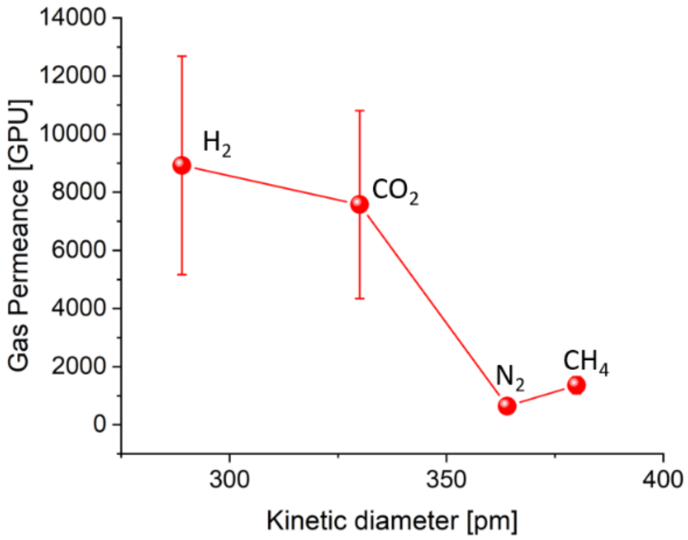

Figure S7. Control experiment for probing thermal gasification. Gas permeance data as a function of kinetic diameter for a control experiment probing thermal gasification in comparison to photonic gasification. Gasification of oxygen clusters was carried out by heating at 500°C for 1 h after 80 °C oxidation.

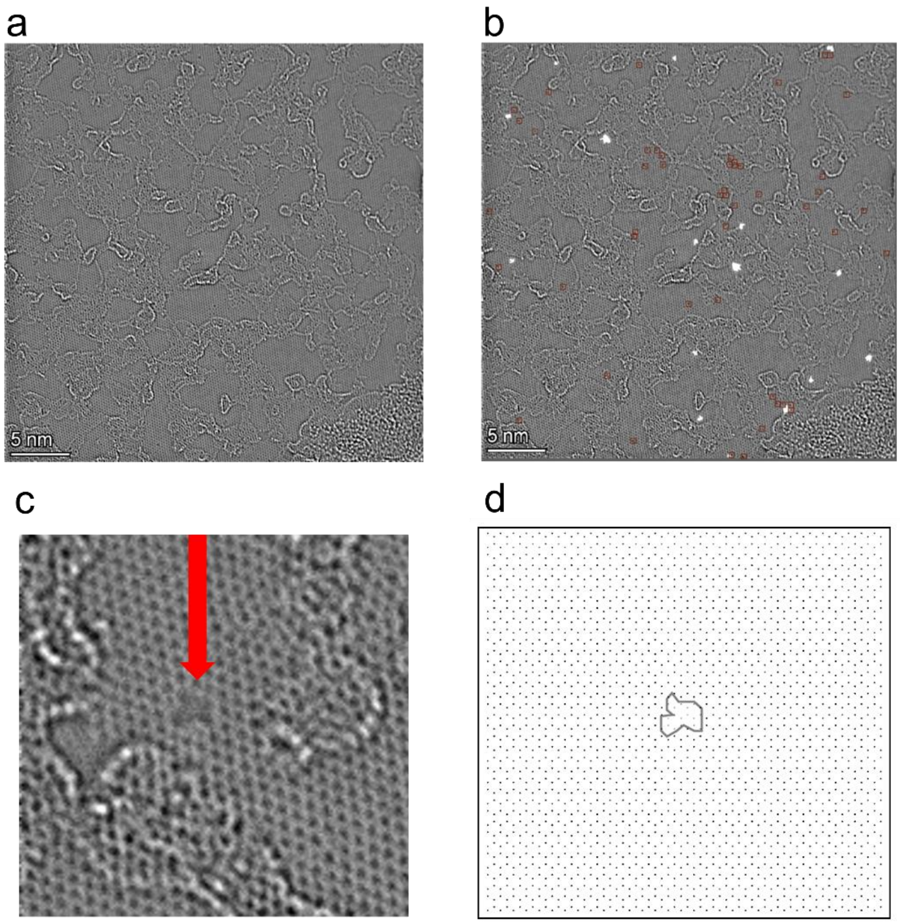

Figure S8. AC-HRTEM method to estimate pore size and density adapted from the reference[1]. a) As-obtained HRTEM image. b) Pores are highlighted in white. Monovacancies are highlighted in red squares. Number of missing carbon atoms was quantified by fitting a graphene lattice inside the pores.

Pores touching contaminations were considered in the pore density estimation. c) Pore identification (red arrow). d) Lattice fitting of identified pore in c. The initial number of missing carbon atoms is predicted by the coverage of the pore area on the lattice. Then, the number of carbon atoms forming the contour line is then further used to affine the prediction of the number of missing carbon atoms of the pore.

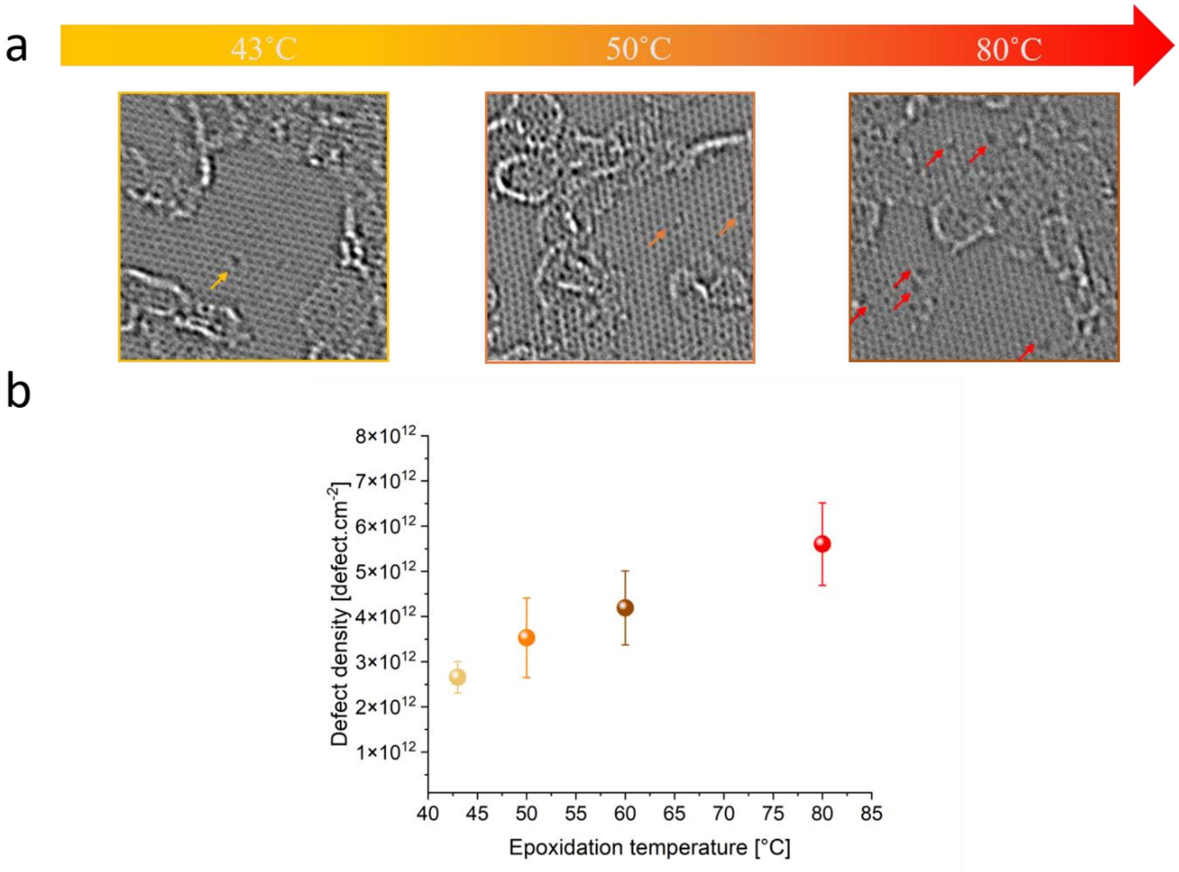

Figure S9. Defect density evolution as a function of oxidation temperature. a) AC-HRTEM images of defects formed by 5s light gasification of oxygen clusters at different ozone functionalisation temperature. Arrows highlight defects generated upon gasification. b) Density of vacancy defects (including single vacancies) as a function of the oxidation temperature. The error bar is the standard deviation from 10 images.

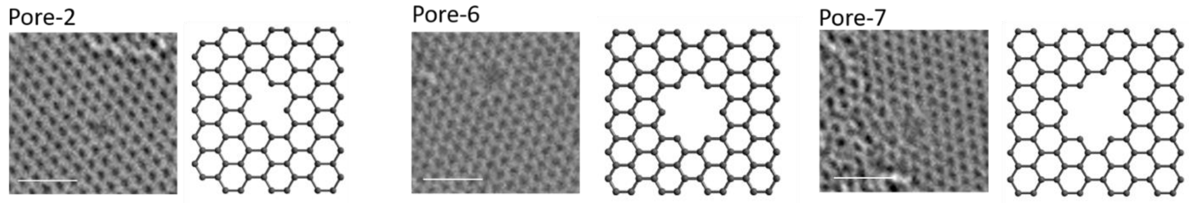

Figure S10. AC-HRTEM images of small non-permeable defects generated by oxidation at 80 ºC O_3 followed by 5s photonic gasification. The scale bar is 1 nm.

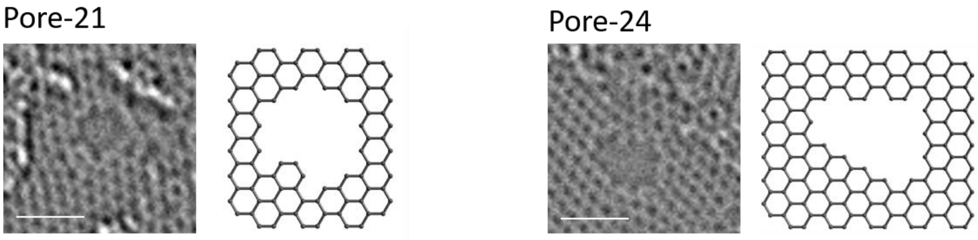

Figure S11. AC-HRTEM images of large non-selective pores generated by oxidation at 80 ºC O_3 followed by 5s photonic gasification. The scale bar is 1 nm.

Supplementary Note S2

Investigation of mass transport in confined spaces by screening of gas permeable pores. Computing gas permeance from the vast possibilities of graphene pores with varying number of missing carbon atoms and varying degree of pore edge functionalization is a lengthy and extremely expensive task by molecular dynamics (MD) simulation. To rapidly screen pores, we relied on the pore screening tool reported elsewhere.[2] Using Avogadro software (version 1.2.0), each pore was represented using its van der Waals (vdW) gap. To estimate the most favourable configuration for pore edge functional groups, the functional groups were relaxed under the universal force field (UFF) while fixing the carbon atoms of the graphene lattice. The resulting vdW gap yielded a pore limiting diameter (PLD) (Figure S12). Following this, two different approaches were used and cross compared to assess permeable pore-gas pairs. In the first screening method (PLD screening method), kinetic diameter of each gas molecules was compared to pore PLD, defined as the diameter of the biggest sphere that can fit within the vdW gap of the pore (Fig. S12). If kinetic diameter is smaller than the PLD, gas molecule is assumed to permeate through the pore. To cross-confirm the first approach, relaxed gas molecules by UFF force field were placed inside the pores (vdW screening method). If the molecule can fit within the pore without overlap with vdW space occupied by the pore edge functional groups, the molecule is assumed to permeate through the pore. Similarly, if the molecule did not fit within the pore without overlap with the vdW space of the pore edge functional group, the pore is assumed to be impermeable for that molecule. This method has been validated for unfunctionalized (carbon passivated) pores using MD simulation in the literature.[2] For oxygen-functionalized pores, while this method has not been validated by MD simulations, it provides insight whether the pore in question would be permeable or not.

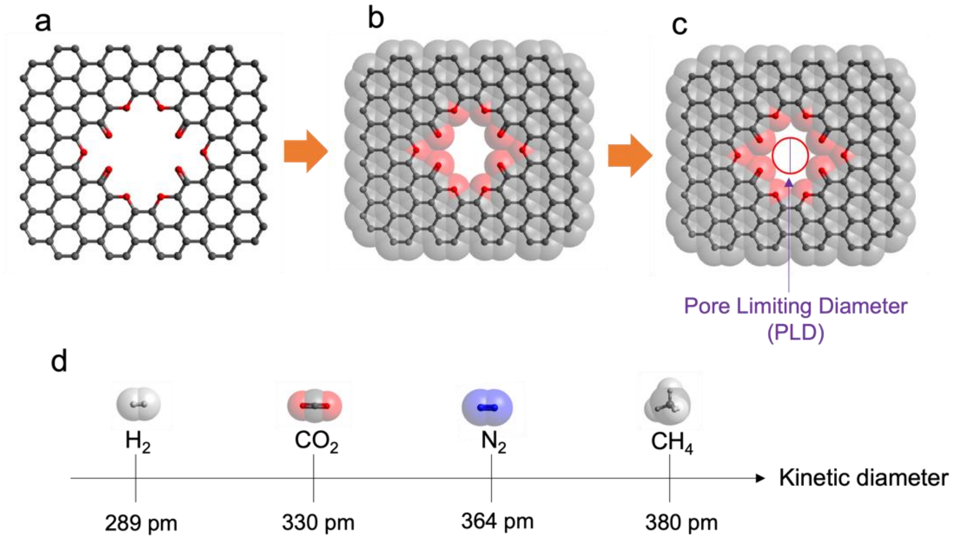

Figure S12. Geometric metrics used on the side of the vdW-screening method. a) Representation of pore-16, decorated by oxygen-functional groups. b) van der Waals representation of the pore in (a). The pore gap is the space depicted by white color at the centre of the pore. c) Estimation of the pore limiting diameter (PLD) of pore. For this, we fitted the largest possible sphere within the vdW gap (in red). The diameter of the sphere represents the PLD.

To identify permeable pores over a broad range of missing carbon atoms, we considered six set of pores, i.e. pores with 6, 7, 8, 10, 13 and 16 missing carbon atoms. The geometry of carbon-passivated pores, without any functional group, were taken directly from AC-HRTEM imaging in this study and from the most probable pore geometry from the isomer catalogue problem reported by Strano and coworkers.[3]

Calculation of PLD based on the functionalization of the pore: The knowledge of the exact functionalization of a pore is a core challenge of the field, given the lack of experimental methods to directly image functional groups at the Å-scale pores. Still XPS provided an important clue, i.e., terminal groups were among ether, epoxy and semiquinone. Given that the core of the cluster, yielding the pore, is made of ether, and given that semiquinone can only exist at the pore edge, we only considered ether and semiquinone to decorate pore edge. Several pore geometries were generated by fully passivating the pore edge with oxygen and varying the number of semiquinone groups (from 1 up to 4) to satisfy the experimental data on the ratio of ether/semiquinone (6.7%).[4]

For each geometry, both vdW screening methods were used to predict whether such pore would be permeable to H_2, CO_2, and CH_4. If any of the pore geometry (resulting from the choice of functionalization) for a given pore skeleton (number of missing carbon size), was permeable to a given gas, it was assumed that the pore skeleton would be permeable to that gas. Resulting analyses is shown in Fig. S13-S24.

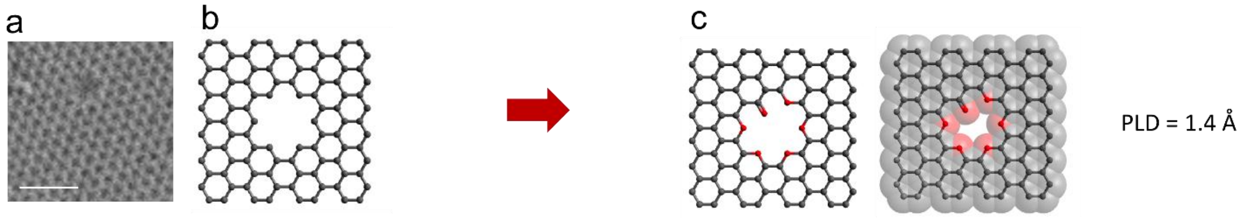

Figure S13. Analysis for pore-6. a) AC-HRTEM imaging of 6 missing carbon atoms. Scale bar is 1 nm. b) Associated pore, simulated in Avogadro. c) Left: estimated oxygen passivation. Right: vdW representation of passivated pore.

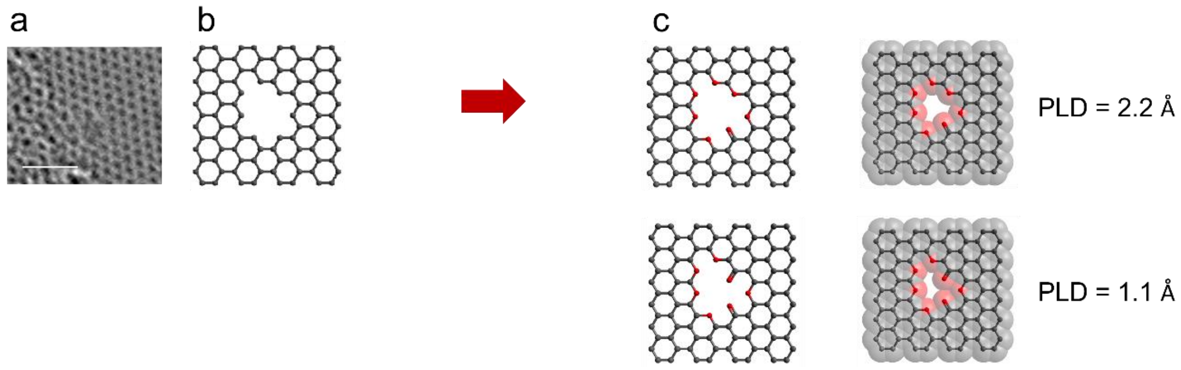

Figure S14. Analysis for pore-7. a) AC-HRTEM imaging of 7 missing carbon atoms. Scale bar is 1 nm. b) Associated pore, simulated in Avogadro. c) Left: estimated oxygen passivation. Right: vdW representation of passivated pore.

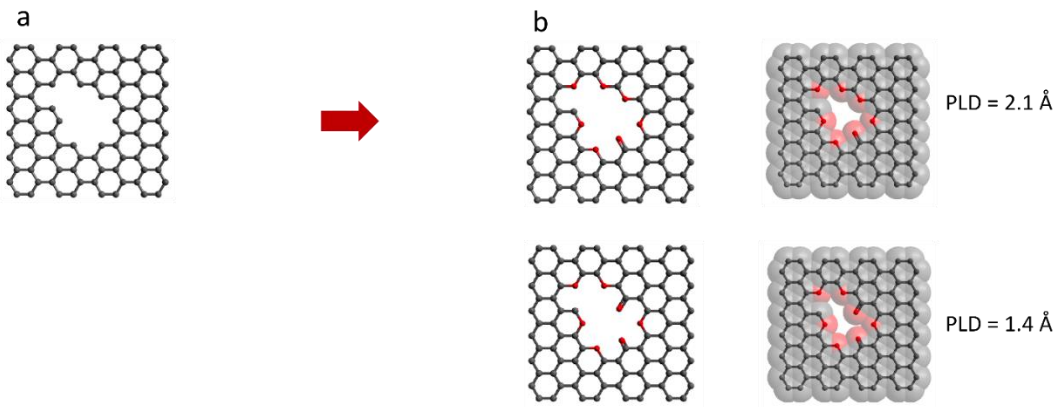

Figure S15. Analysis for pore-8. a) Pore geometry of the most probable isomer for 8 missing carbon, simulated in Avogadro.[3] b) Passivation estimation of pore-8. Each row is associated to a number of semiquinone passivation. Left: estimated oxygen passivation of pore-8. Right: vdW representation of passivated pore.

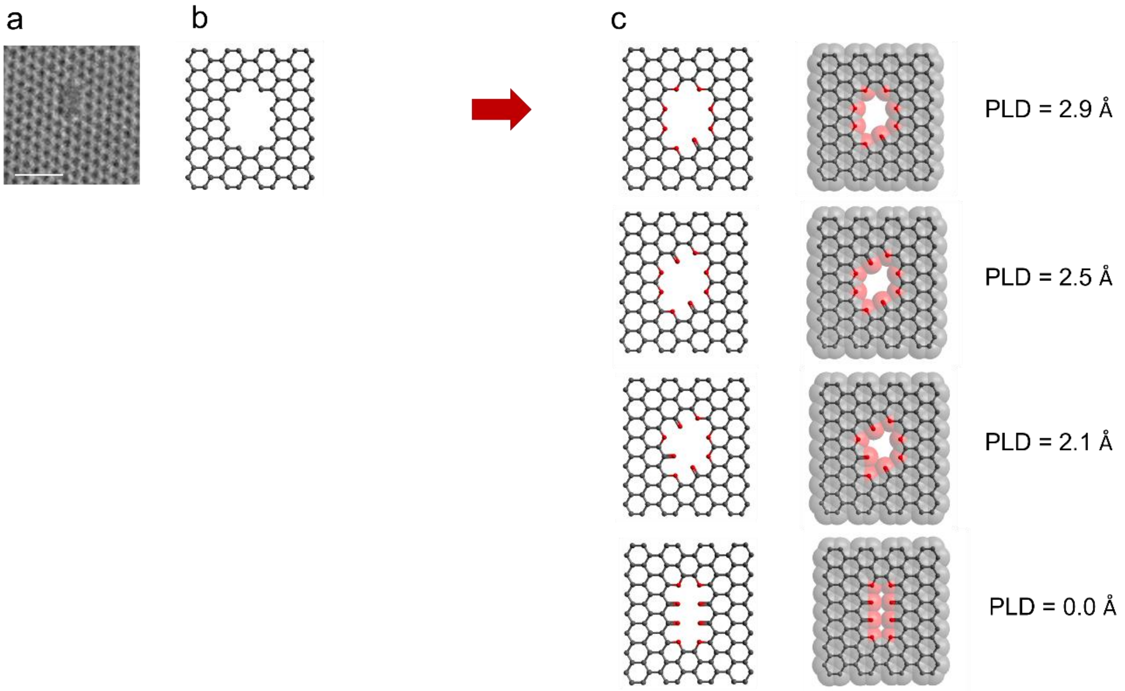

Figure S16. Analysis for pore-10. a) AC-HRTEM imaging of 10 missing carbon atoms at 80 kV. Scale bar is 1 nm. b) Associated pore, simulated in Avogadro. c) Passivation estimation of pore-10. Each row is associated to a number of semiquinone passivation. Left: estimated oxygen passivation of pore-10. Right: vdW representation of passivated pore.

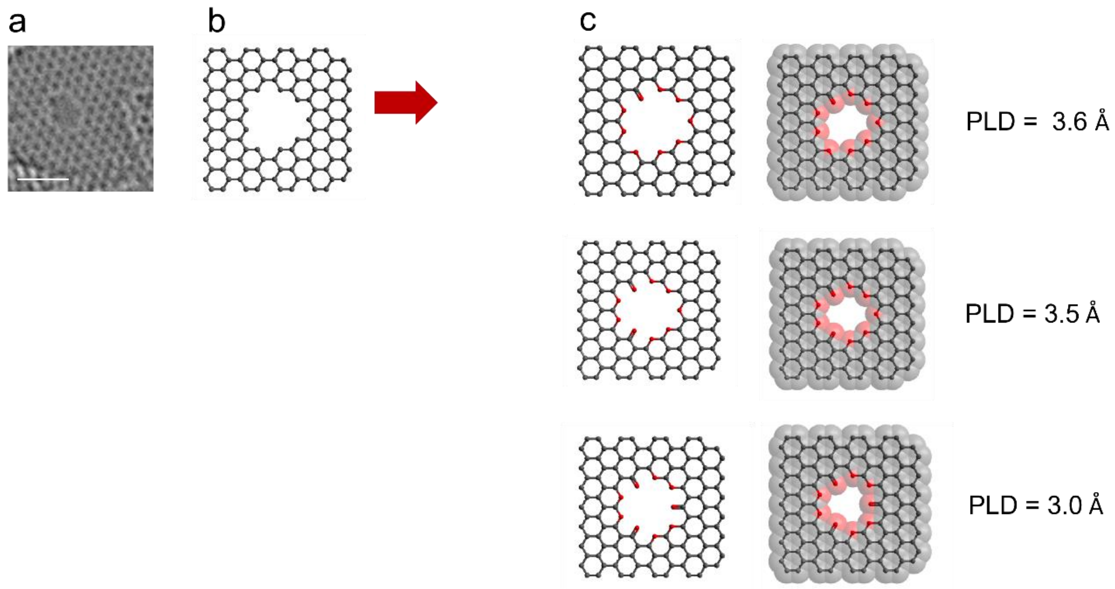

Figure S17. Identification of pore-13. a) AC-HRTEM imaging of 13 missing carbon atoms at 80 kV. Scale bar is 1 nm. b) Associated pore, simulated in Avogadro. c) Passivation estimation of pore-13. Each row is associated to a number of semiquinone passivation. Left: estimated oxygen passivation of pore-13. Right: vdW representation of passivated pore.

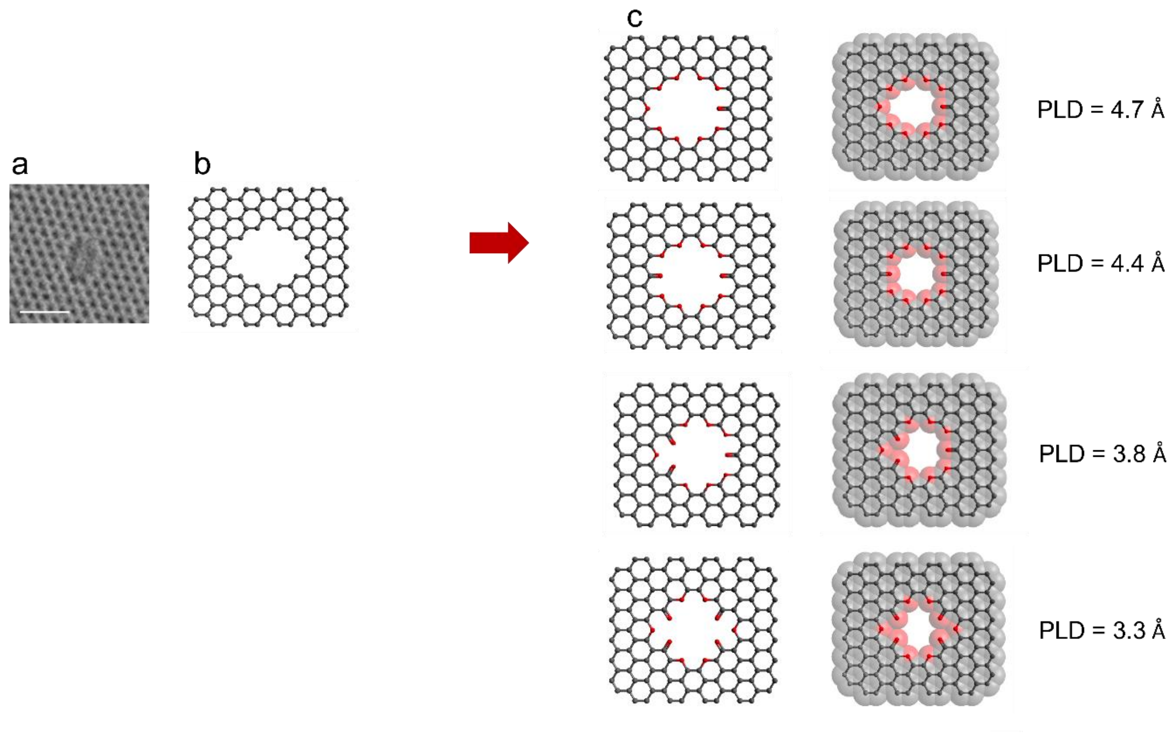

Figure S18. Analysis for pore-16. a) AC-HRTEM imaging of 16 missing carbon atoms at 80 kV. Scale bar is 1 nm. b) Associated pore, simulated in Avogadro. c) Passivation estimation of pore-16. Each row is associated to a number of semiquinone passivation. Left: estimated oxygen passivation of pore-16. Right: vdW representation of passivated pore.

Based on the comparison between the PLD and the kinetic diameter of the gas molecule, H_2 permeates through pore-10, CO_2 through pore-13 and both N_2 and CH_4 through pore-16. Summary of the PLD method is depicted in the table below:

Table S3: Permeability assessment of gas molecules by comparing PLD and kinetic diameter of gas molecules. The assessment is an approximation by comparing the PLD of O-functionalized pores and kinetic diameter of gas molecules.

Gas molecule	Estimated permeability region in terms of missing carbon atoms
H_2	≥10
CO_2	≥13
CH_4	≥16
N_2	≥16

To further strengthen the permeability assessment, a second method mentioned above (vdW screening method), considering the geometry of gas molecule was employed. To cross-confirm the first approach, relaxed gas molecules by UFF force field were placed inside the pores. If the molecule fitted within the pore without overlap with vdW space occupied by the pore edge functional groups, the molecule is assumed to permeate through the pore. Similarly, if the molecule did not fit within the pore without overlap with the vdW space of the pore edge functional group, the pore is assumed to be impermeable for that molecule. However, given the vdW radius of N atom (155 pm) comparing to C atom (170 pm), such method is inefficient to predict permeation of N_2. Assuming that gas molecules will permeate in a perpendicular fashion to the pore, the molecular limiting diameter (MLD), defined as the biggest diameter of the smallest molecule cross section, would be bigger for CO_2 than N_2 and thus, leading to inaccurate prediction of N_2 permeation. Therefore, in this study, only H_2, CO_2 and CH_4 are considered.

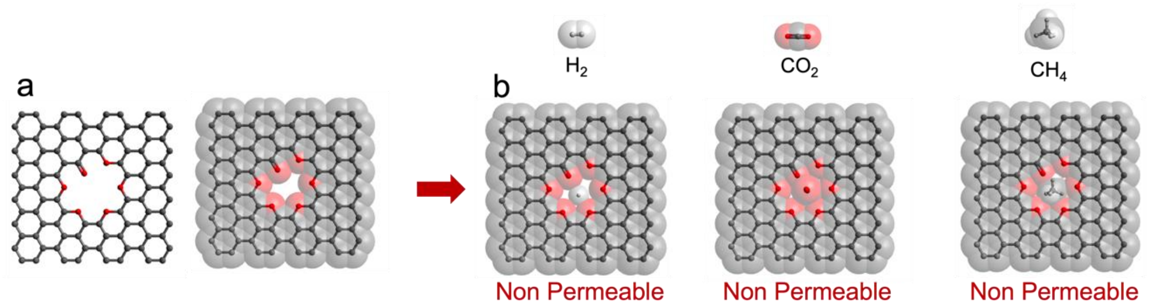

Figure S19. Permeation assessment of pore-6. a) Left: passivated pore simulated in Avogadro. Right: vdW representation of pore on the right. b) vdW screening method. Top: vdW representation of H_2, CO_2, CH_4. Each column is associated to a gas molecule from left to right: H_2, CO_2 and CH_4. Permeability of the pore/gas pair is depicted under each pair.

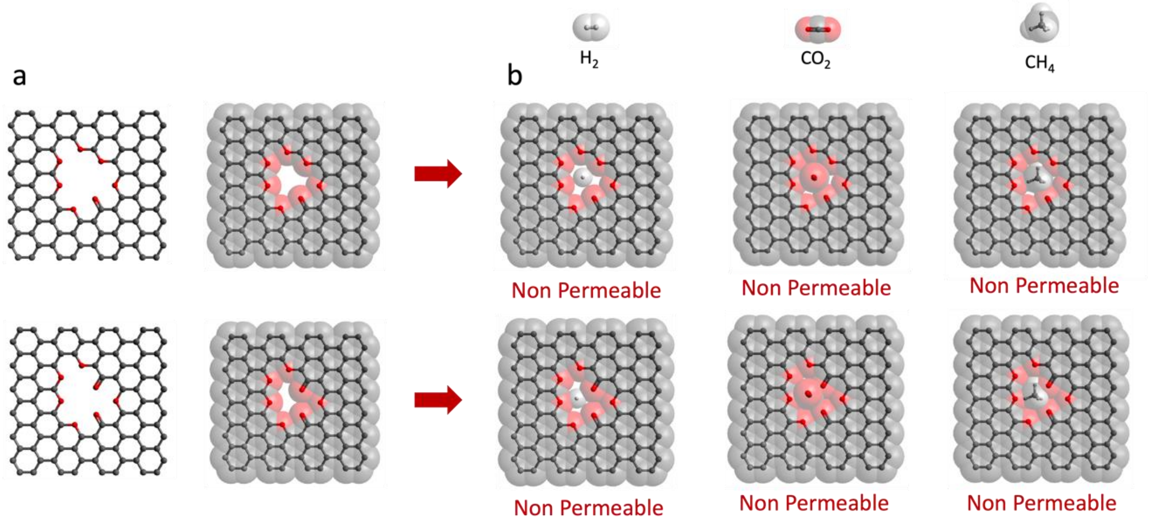

Figure S20. Permeation assessment of pore-7. a) Left: passivated pore simulated in Avogadro. Right: vdW representation of pore on the right. b) vdW screening method. Top: vdW representation of H_2, CO_2, CH_4. Each column is associated to a gas molecule from left to right: H_2, CO_2 and CH_4. Permeability of the pore/gas pair is depicted under each pair.

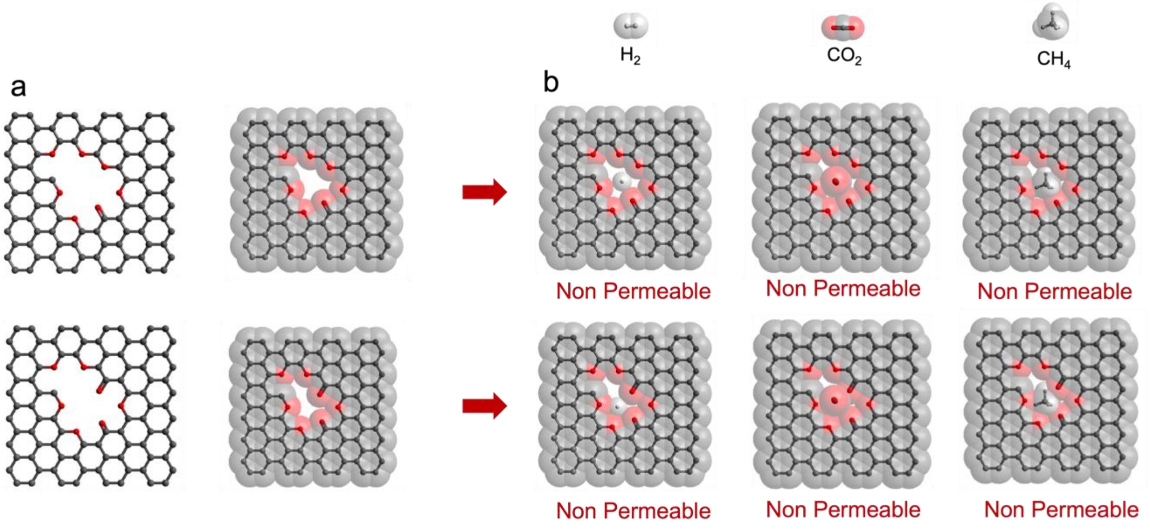

Figure S21. Permeation assessment of pore-8. a) Left: passivated pore simulated in Avogadro. Right: vdW representation of pore on the right. b) vdW screening method. Top: vdW representation of H_2, CO_2, CH_4. Each column is associated to a gas molecule from left to right: H_2, CO_2 and CH_4. Permeability of the pore/gas pair is depicted under each pair.

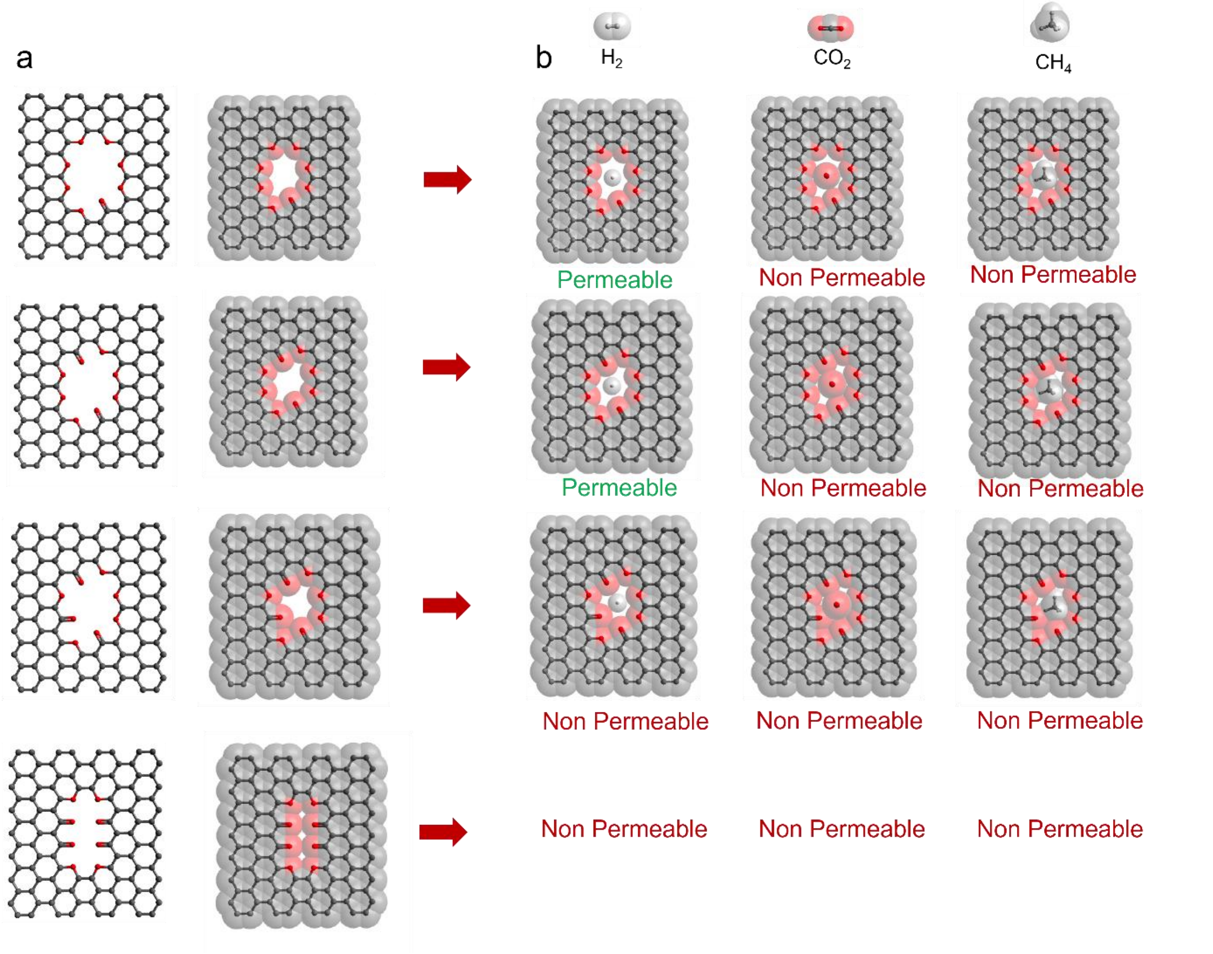

Figure S22. Permeation assessment of pore-10. a) Left: passivated pore simulated in Avogadro. Right: vdW representation of pore on the right. b) vdW screening method. Top: vdW representation of H_2, CO_2, CH_4. Each column is associated to a gas molecule from left to right: H_2, CO_2 and CH_4. Permeability of the pore/gas pair is depicted under each pair.

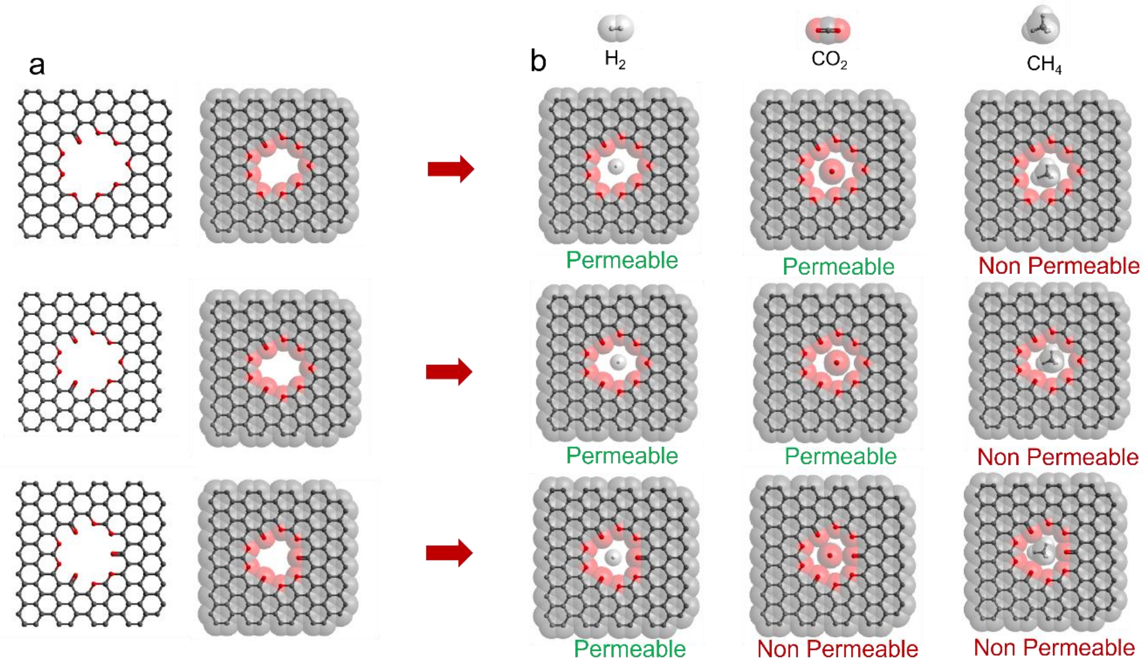

Figure S23. Permeation assessment of pore-13. a) Left: passivated pore simulated in Avogadro. Right: vdW representation of pore on the right. b) vdW screening method. Top: vdW representation of H_2, CO_2, CH_4. Each column is associated to a gas molecule from left to right: H_2, CO_2 and CH_4. Permeability of the pore/gas pair is depicted under each pair.

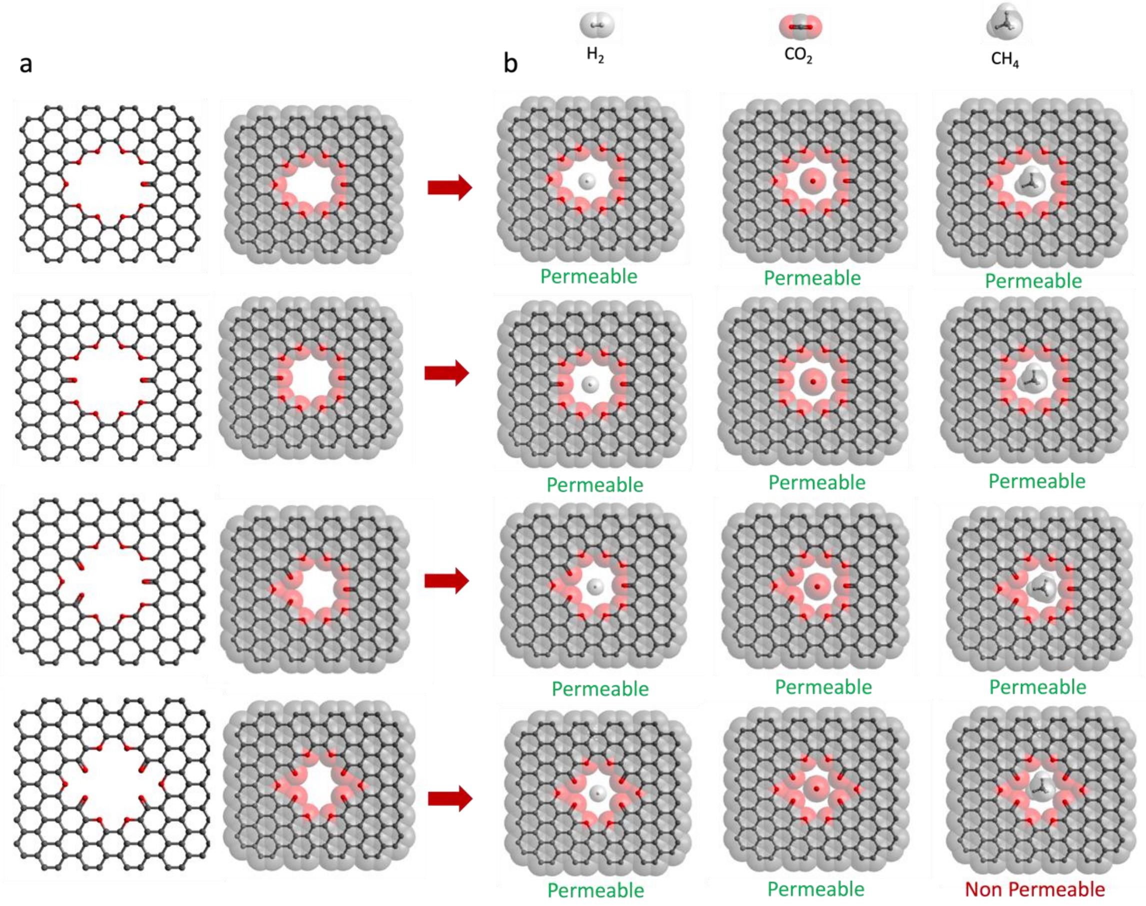

Figure S24. Permeation assessment of pore-16. a) Left: passivated pore simulated in Avogadro. Right: vdW representation of pore on the right. b) vdW screening method. Top: vdW representation of H_2, CO_2, CH_4. Each column is associated to a gas molecule from left to right: H_2, CO_2 and CH_4. Permeability of the pore/gas pair is depicted under each pair.

Based on this second method, H_2 is expected to permeate from pore-10 and thus, suggesting that smaller pores are impermeable. CO_2 starts to permeate only from pore-13, while CH_4 is expected to permeate from pore-16 only. This prediction is consistent with the PLD screening method using kinetic diameter. Given the fact that vdW screening method will not be effective to estimate permeation of N_2 (vdW cross-section of N_2 is smaller than that of CO_2), the prediction of N_2 permeation probability was based using the kinetic diameter method only.

Given that both screening methods yield similar results, a table summarizing permeability region for each gas molecule is depicted below:

Table S4. Estimated gas permeability regime as a function of the number of missing carbon atoms in the pore.

Gas molecule	Permeable pores in terms in number of missing carbon atoms
H_2	≥10
CO_2	≥13
N_2	≥16
CH_4	≥16

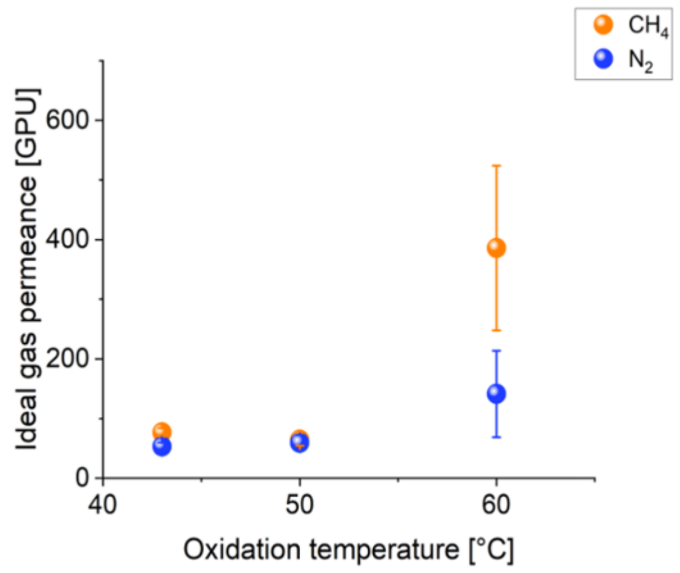

Figure S25. CH_4 and N_2 permeation across porous graphene for several oxidation temperatures followed by 5 s light gasification. The error bar represents standard deviation from 3 samples.

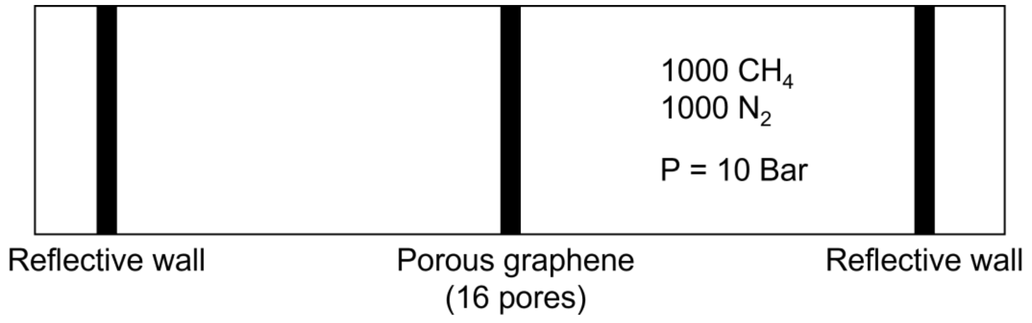

Figure S26. Schematic for MD simulations. The system is used to investigate CH_4/N_2 mixture permeation across graphene pores.

Supplementary Note S3

Simulation of CH_4 and N_2 permeation across pore with 13 missing carbon atoms

Given the small size of the electronic density gap, pore-13, depicted in Figure S27, was impermeable for both CH_4 and N_2. This is greatly consistent with screening methods from above, where it was estimated that both CH_4 and N_2 would start permeating from pore-16.

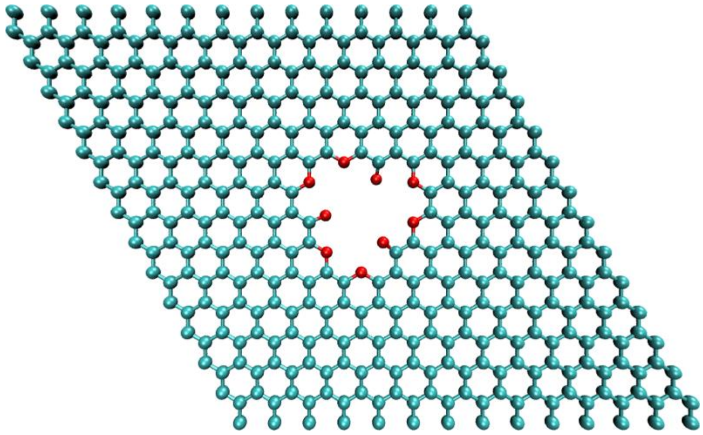

Figure S27. Structure of Pore-13. The pore edge is terminated by ether and semiquinone groups.

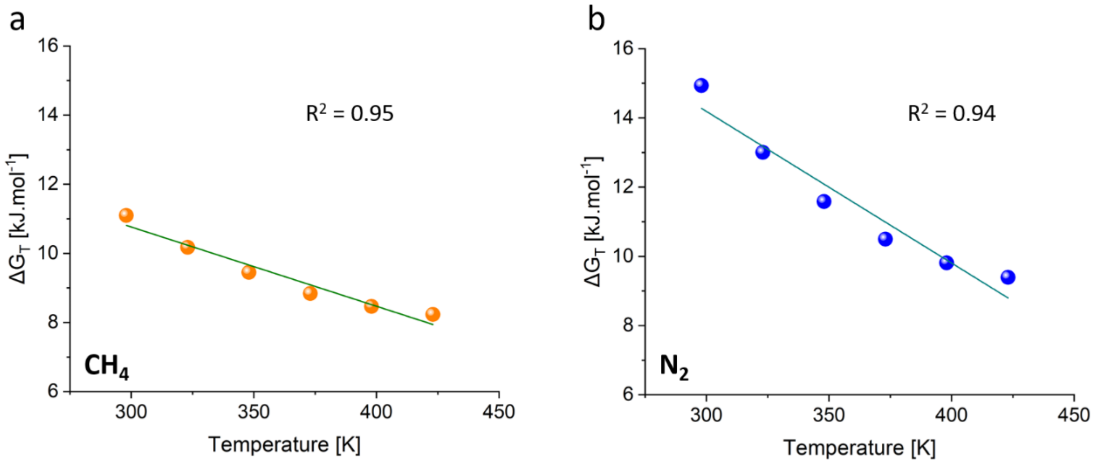

Figure S28. Linear fit of pore translocation free energy (ΔG_T) across Pore-16 as a function of temperature. a) CH_4. b) N_2.

Supplementary Note S4

Enthalpy of pore translocation

Based on the suitable linear fit of ΔG_T for both CH_4 and N_2 with temperature, both ΔH_T and ΔS_T can be assumed to be independent of temperature. Based on this assumption the subsequent equation can be used for each simulation window:

$$\Delta G = \Delta H - T \cdot \Delta S$$

Based on the linear regression, ΔH as a function of z could be estimated (Fig. S28).

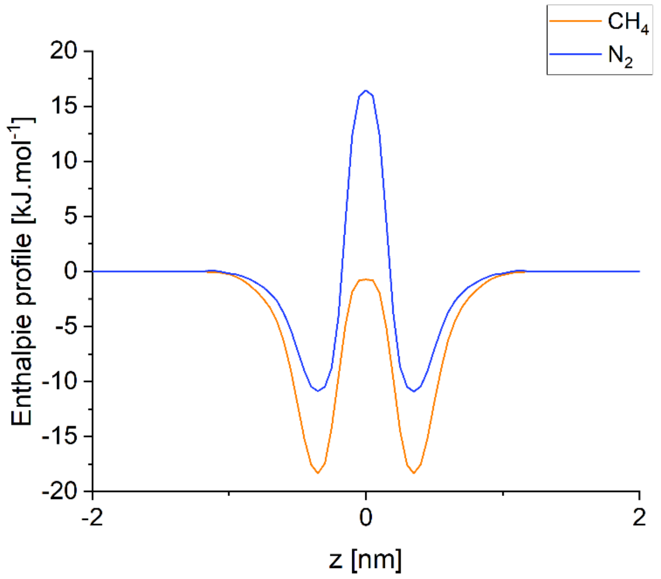

Figure S29. Enthalpy profile. The profile is shown along z axis for both CH_4 and N_2 permeation across pore-16.

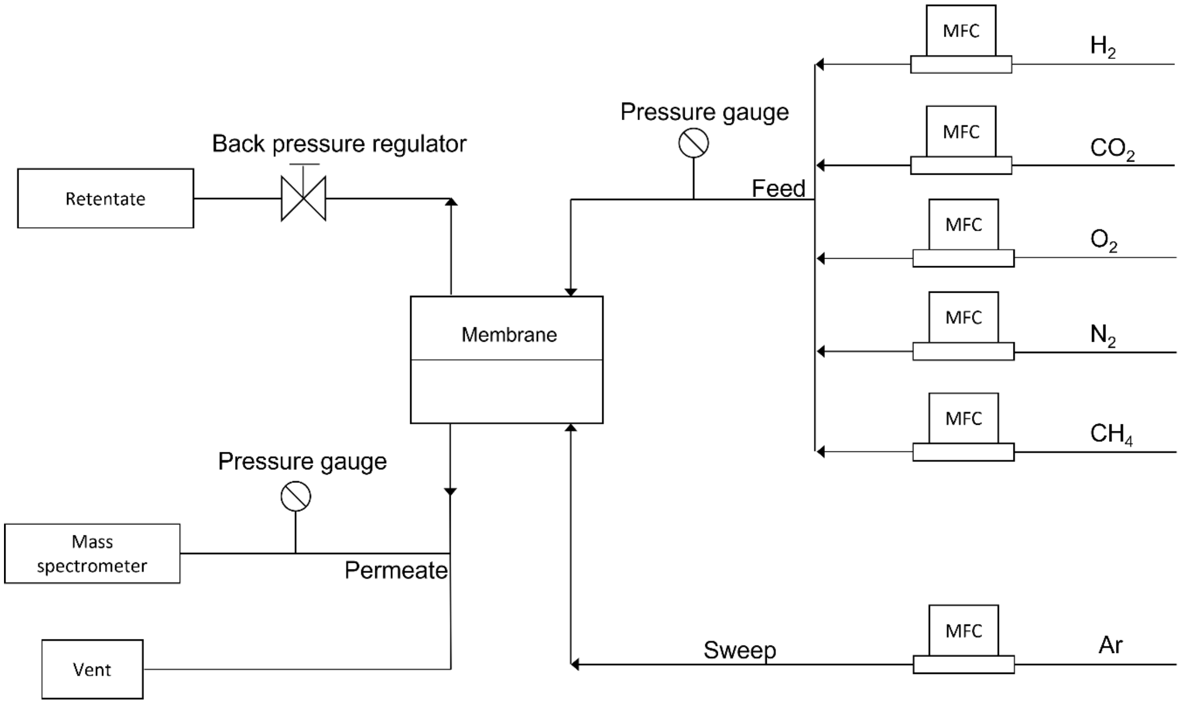

Figure S30. Schematic of the gas permeation set-up.

References

(1) Hsu, K. J.; Villalobos, L. F.; Huang, S.; Chi, H. Y.; Dakhchoune, M.; Lee, W. C.; He, G.; Mensi, M.; Agrawal, K. V. Multipulsed Millisecond Ozone Gasification for Predictable Tuning of Nucleation and Nucleation-Decoupled Nanopore Expansion in Graphene for Carbon Capture. *ACS Nano* **2021**, *15* (8), 13230–13239. https://doi.org/10.1021/acsnano.1c02927.

(2) Bondaz, L.; Chow, C.-M.; Karnik, R. Rapid Screening of Nanopore Candidates in Nanoporous Single-Layer Graphene for Selective Separations Using Molecular Visualization and Interatomic Potentials. *J Chem Phys* **2021**, *154* (18), 184111. https://doi.org/10.1063/5.0044041.

(3) Govind Rajan, A.; Silmore, K. S.; Swett, J.; Robertson, A. W.; Warner, J. H.; Blankschtein, D.; Strano, M. S. Addressing the Isomer Cataloguing Problem for Nanopores in Two-Dimensional Materials. *Nat Mater* **2019**, *18* (2), 129–135. https://doi.org/10.1038/s41563-018-0258-3.

(4) Lee, W. C.; Ronghe, A.; Villalobos, L. F.; Huang, S.; Dakhchoune, M.; Mensi, M.; Hsu, K. J.; Ayappa, K. G.; Agrawal, K. V. Enhanced Water Evaporation from Å-Scale Graphene Nanopores. *ACS Nano* **2022**, *16* (9), 15382–15396. https://doi.org/10.1021/acsnano.2c07193.
